# Single-cell mapping of diabetic foot ulcers: mechanistic axes and therapeutic targets

**DOI:** 10.3389/fmed.2026.1910324

**Published:** 2026-08-13

**Authors:** Haoyu Zhang, Minjia Tao, Chengkai Yang, Ahmedur Rahman Sabuj, Mridul Kanti Sarker, Cheng Wang

**Affiliations:** 1 Department of Burns, Beijing Jishuitan Hospital, Capital Medical University, Beijing, China; 2 National Institute of Burn and Plastic Surgery, Dhaka, Bangladesh; 3 Peking University Fourth School of Clinical Medicine, Beijing, China

**Keywords:** angiogenesis, cellular senescence, diabetic foot ulcer, keratinocytes, single-cell RNA sequencing, spatial transcriptomics, wound healing

## Abstract

Diabetic foot ulcer (DFU) is a heterogeneous chronic wound in which the orderly sequence of inflammation, repair, and remodeling is replaced by persistent non-healing. Although bulk transcriptomic profiling and histopathology have revealed inflammatory imbalance and protease dysregulation, they cannot resolve the cell states, intercellular communication, and spatial niches that sustain chronicity or define residual reparative capacity. Recent single-cell and spatial studies are redefining DFU as a dynamic cellular ecosystem comprising epithelial, stromal, immune, vascular, and adnexal populations with distinct states and transitions. This review synthesizes these emerging datasets and proposes a mechanistic framework in which DFU chronicity is driven by four interrelated domains: epidermal arrest, characterized by stress-adapted basal keratinocytes that fail to complete activation-to-differentiation programs; immune-stromal lock-in, maintained by chemokine-mediated leukocyte recruitment, protease-biased matrix turnover, and defective resolution of inflammation; vascular dysfunction with neurovascular uncoupling, marked by endothelial stress, impaired angiogenesis, hypoperfusion, hypoxia, and loss of neurovascular and eccrine-associated homeostatic support; and proliferative arrest with senescence burden, in which persistent p21-associated cell-cycle arrest and senescent cell accumulation limit regenerative capacity while amplifying inflammatory and proteolytic signaling. Together, these cellular and spatial features provide a unifying explanation for DFU non-healing and highlight candidate biomarkers, therapeutic opportunities, and translational priorities required to convert descriptive atlases into actionable interventions.

## Graphical abstract

**Figure:**
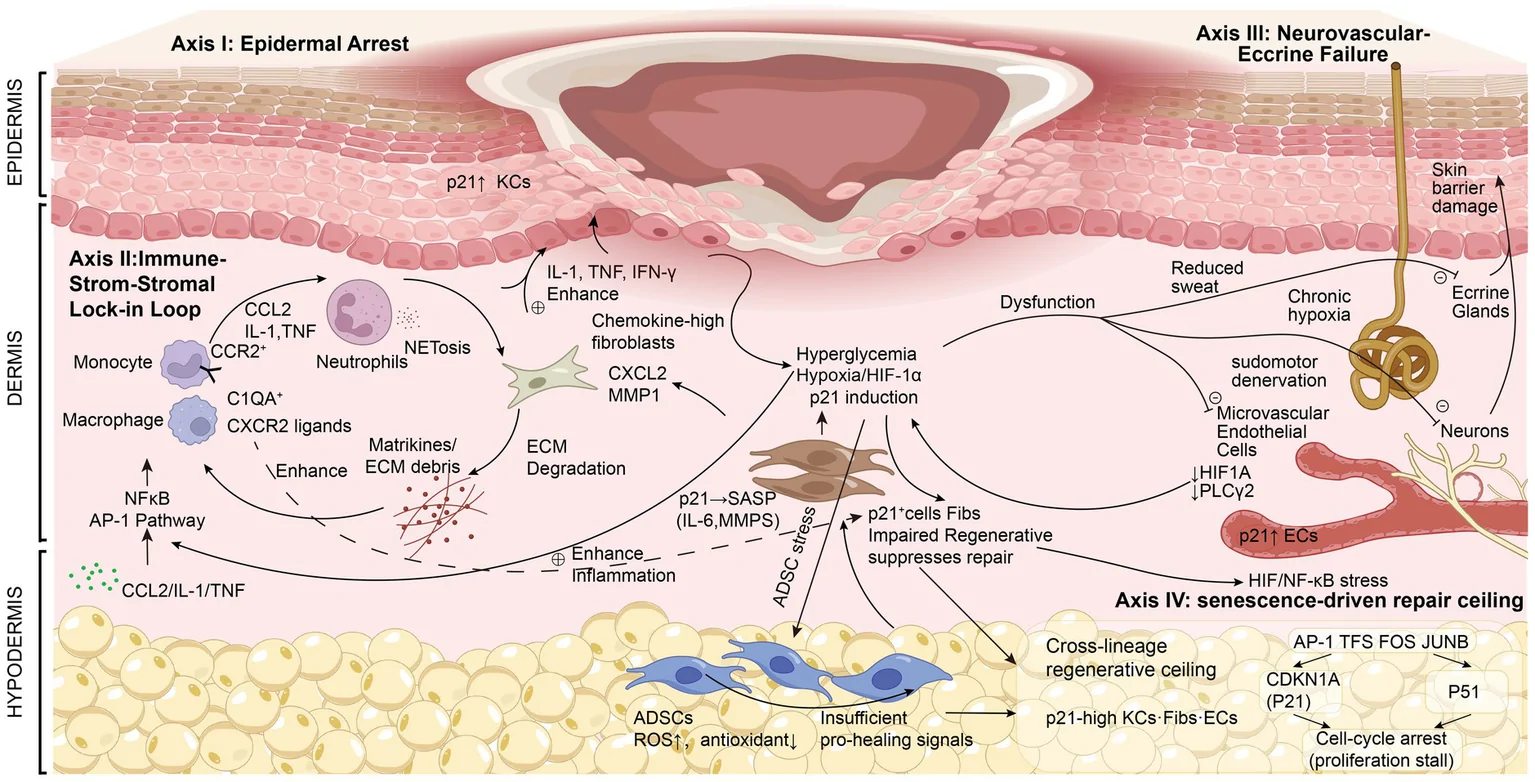

## 1 Introduction

Diabetic foot ulcers (DFUs) are chronic wounds wherein the transition from inflammation to repair is disrupted. DFUs are characterized by persistent inflammation, elevated protease activity, impaired extracellular matrix (ECM) remodeling, reduced perfusion, and diminished regenerative capacity. The lifetime risk of developing a DFU is 19–34%, with a 65% recurrence rate within 3–5 years. The five-year mortality rate is 50–70%, with 20% of cases requiring amputation (1–3). DFUs develop in the context of systemic comorbidities, where cardiometabolic, vascular, and infectious burdens limit healing.

DFU nonhealing is not a uniform biological condition. Ulcers with the same clinical diagnosis differ in aspects of neuropathic burden, ischemic impairment, microbial ecology, depth, biomechanical loading, renal disease, and systemic inflammation. Despite standard interventions, these differences result in distinct healing trajectories (e.g., debridement, offloading, antimicrobial therapy, and revascularization). This heterogeneity is not merely random clinical variability. Wound closure requires multiple coupled processes to occur simultaneously, such as adequate perfusion, control of bioburden, resolution of inflammation, and effective matrix remodeling and re-epithelialization. Variation across these axes means that the dominant constraint on closure differs among patients and may shift across phases within the same wound. In practice, generalized pro-healing therapies often fall short unless guided by a state-aware assessment of which cellular programs the wound microenvironment allows to function or suppresses. Single-cell DFU atlases have begun to formalize this heterogeneity as recurrent configurations of cell states rather than simple differences in bulk inflammatory intensity. Theocharidis et al. (4) constructed a dataset of 174,962 cells from 54 samples, which comprised DFU tissue, foot and forearm skin, and peripheral blood. They stratified DFU by 12-week outcome, enabling direct comparison between wounds that progressed to healing and those that remained clinically unchanged. Throughout this review, we use “healing DFUs” to refer to ulcers that healed within the specified follow-up period and “non-healing DFUs” to refer to ulcers that failed to heal or remained clinically unchanged according to the definitions of the original studies.

Single-cell and spatial approaches make DFU biology more mechanistically resolvable, but they do not by themselves establish causality (5). Bulk assays obscure cellular diversity by averaging mixed populations, whereas single-cell and spatial methods attribute signals to specific cell states, recover rare transitional populations, and preserve or reconstruct spatial relationships. Ligand–receptor inference, pseudotime reconstruction, and RNA velocity analysis generate testable hypotheses regarding intercellular communication and state transitions rather than direct evidence of signaling or temporal progression. Choi et al. (6) showed that debridement-derived human DFU tissue yields high-quality single-cell profiles, supporting serial molecular assessment with limited additional tissue collection. These approaches therefore support mechanistic hypothesis generation and targeted validation in DFU.

To define the evidentiary scope of this mechanistic synthesis, we focused on human skin and wound datasets that directly inform DFU biology or provide relevant acute-wound and normal-skin comparators. Core evidence was drawn from single-cell RNA sequencing (scRNA-seq), single-nucleus RNA sequencing where available, and spatial transcriptomic studies of DFU tissue, diabetic foot or forearm skin, acute wound edges, wound-edge biopsies, wound-bed or debridement specimens, paired peripheral blood, and normal skin controls, provided that cell-type or cell-state annotations and sampling context were sufficiently described (4, 6–8). Studies centered on non-cutaneous tissues, non-DFU chronic wounds without a diabetic-foot comparator, datasets lacking interpretable cell-state annotations or sampling-site information, and reports with insufficient clinical grouping for outcome-specific interpretation were not used as core evidence for DFU-specific cell-state programs. Datasets without healing outcome annotation were considered for cell-state and niche biology but were not used to support claims about differences between healing DFUs and non-healing DFUs. These criteria do not constitute a systematic-review protocol; they define the evidence base used to compare recurrent cell states, spatial niches, and axis-level mechanisms across complementary datasets.

The evidentiary strength of the studies reviewed here depends on the biological system and study design. Human single-cell and spatial studies define cell states, spatial organization, and associations with healing outcomes, but their largely cross-sectional design does not establish causality (9). Rodent models permit controlled genetic and pharmacological perturbation and provide mechanistic or therapeutic proof-of-concept. Their translation to human DFU is limited by species-specific skin architecture, the substantial contribution of wound contraction—particularly in unsplinted models—and the incomplete representation of chronic ischemia, neuropathy, infection, aging, renal disease, and multimorbidity (10). *In vitro* and *ex vivo* systems isolate pathway-level effects under defined conditions but do not reproduce the complete wound environment. Throughout the text, we therefore identify each study as human, clinical, rodent, *in vitro*, or *ex vivo* and distinguish clinical efficacy from preclinical therapeutic evidence.

Technical differences across datasets were considered when interpreting the consistency of cell-state definitions. Wound-edge biopsies provide clearer compartmental anchoring for the epidermal–dermal interface and local inflammatory niches, whereas debridement-derived specimens are clinically practical and enable serial sampling but may overrepresent wound-bed, necrotic, inflammatory, or granulation-tissue compartments. Sequencing depth, cell capture efficiency, tissue digestion, and viability thresholds can influence the recovery of rare transitional states, fragile endothelial or immune populations, and stress–response programs induced during single-cell suspension preparation. Spatial transcriptomic platforms retain tissue architecture, although spot size and capture resolution may merge keratinocytes, fibroblasts, vascular cells, and immune cells within dense granulation tissue. Accordingly, cross-cohort convergence was interpreted primarily at the level of reproducible cell-state programs, directional trajectory changes, spatial organization, and coupled mechanistic axes, rather than as direct quantitative comparisons of absolute cell proportions (11–13).

Cell-resolved atlases become therapeutically useful when they explain why DFU wounds stall and identify where interventions can alter the outcome. This requires cross-lineage modules that specify the rate-limiting step, the sustaining signals, and the testable intervention node. We organize DFU non-healing around four coupled axes: (1) Epidermal arrest, where basal keratinocytes adopt stress and inflammatory programs and fail to complete productive differentiation; (2) Immune-stromal lock-in, where chemokine-driven leukocyte influx, protease-skewed ECM turnover, and poor resolution reinforce a self-sustaining inflammatory microenvironment; (3) Neurovascular-eccrine failure, where hypoperfusion and neuropathy impair oxygen delivery, neurogenic control, and sweat gland-linked barrier homeostasis; and (4) Proliferative arrest and senescence burden, where persistent p21-associated arrest and senescent-cell accumulation constrain cycling states while maintaining inflammatory and proteolytic outputs. Therefore, we synthesize the literature along these four mechanistic axes, and within each axis, we move from dysregulated cell states to the signaling and microenvironmental processes that sustain them to the specific transition block that defines failure, and finally to matched intervention targets and windows.

## 2 Single-cell-defined axes of DFU non-healing

### 2.1 Axis I—epidermal arrest

Epidermal failure in DFU results from disrupted keratinocyte function and impaired regenerative progression. In acute wounds, basal keratinocytes transition from quiescence to proliferation and differentiation. However, in DFU, this process is arrested, and keratinocytes accumulate in dysfunctional states. Persistent inflammatory signals and metabolic stress contribute to this impairment, preventing differentiation and barrier repair. The epidermis-dermal microenvironment interaction further exacerbates this failure as inflammation and ECM degradation disrupt keratinocyte migration and maturation. Therapeutic strategies aim to restore keratinocyte regenerative capacity by targeting inflammation and epigenetic reprogramming.

#### 2.1.1 Dysregulated keratinocyte states and stalled differentiation

Single-cell studies show that epidermal failure reflects altered keratinocyte state composition and disrupted regenerative progression, not reduced cell abundance. In acute wounds, basal keratinocytes exit quiescence, transiently activate with migratory and proliferative programs, and then differentiate to restore a stratified barrier. Single-cell atlases show that DFU edges are cell-rich but dysfunctional: keratinocytes show injury-response signatures but adopt dysregulated states that block differentiation and barrier restoration (7). The key gap in knowledge here is the mechanisms that sustain these dysfunctional states.

**Figure 1:**
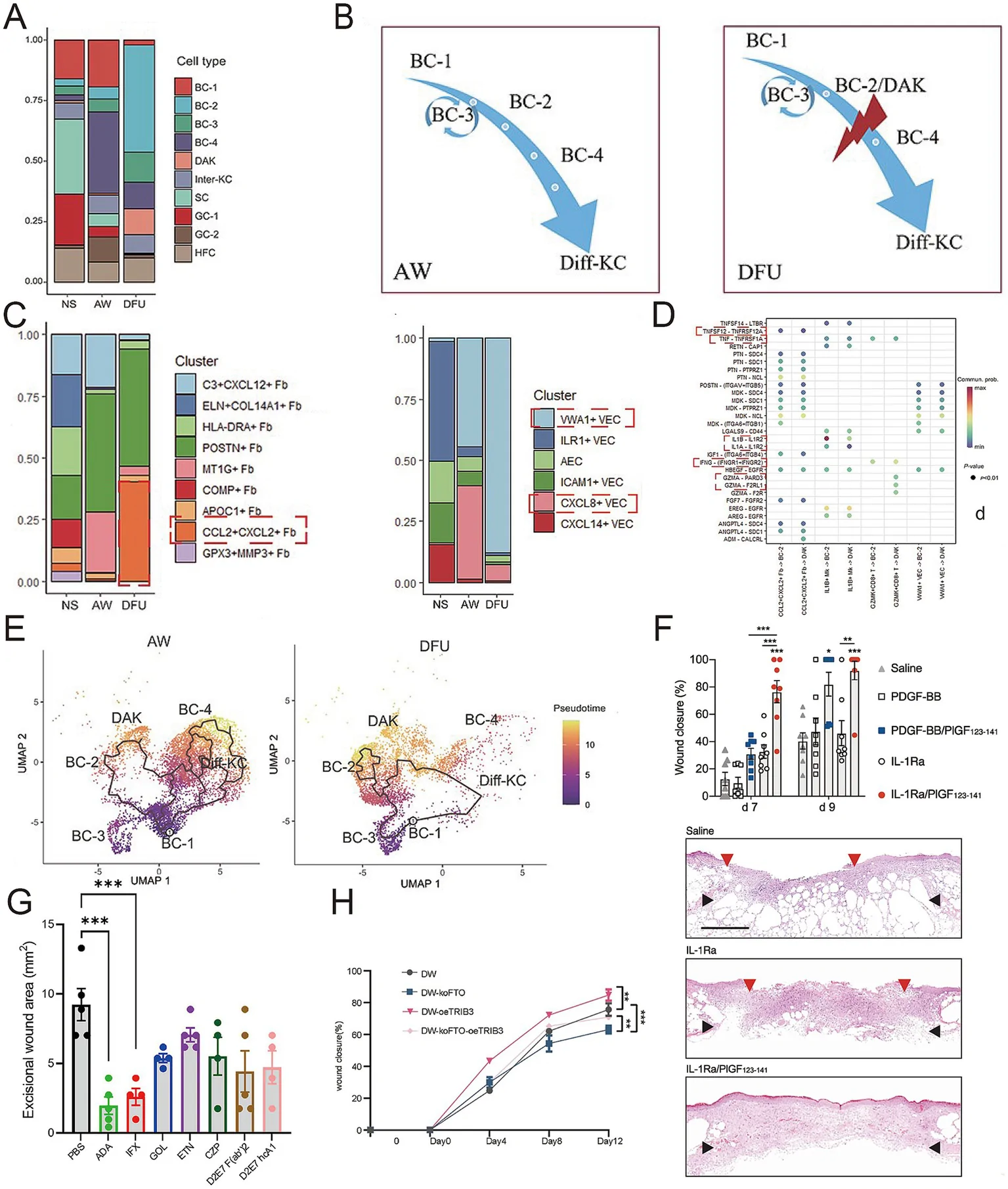
Keratinocyte-state dysregulation in diabetic foot ulcers and preclinical modulation of epidermal repair. **(A)** Relative proportions of keratinocyte subclusters in normal skin, acute wounds, and diabetic foot ulcers (7). **(B)** Schematic representation of keratinocyte differentiation trajectories in acute wounds and diabetic foot ulcers (7). **(C)** Relative proportions of fibroblast and vascular endothelial-cell subclusters in normal skin, acute wounds, and diabetic foot ulcers (7). **(D)** CellChat-predicted ligand–receptor interactions from CCL2^+^CXCL2^+^ fibroblasts, IL1B^+^ macrophages, GZMA^+^CD8^+^ T cells, and VWA1^+^ vascular endothelial cells to basal cell 2 and diabetes-associated keratinocytes in human diabetic foot ulcers (7). **(E)** Pseudotime ordering of keratinocytes in human acute wounds and diabetic foot ulcers, with pseudotime increasing from dark purple to light yellow (7). **(F)** Wound closure and day-9 hematoxylin-and-eosin-stained sections in leptin receptor-deficient diabetic mice treated with saline, unmodified interleukin-1 receptor antagonist (IL-1Ra), matrix-binding interleukin-1 receptor antagonist fused to placenta growth factor 123–141 (IL-1Ra/PlGF_123–141_), unmodified platelet-derived growth factor-BB (PDGF-BB), or matrix-binding platelet-derived growth factor-BB fused to placenta growth factor 123–141 (PDGF-BB/PlGF_123–141_) (*n* = 8 wounds per group). Black arrows indicate the wound edges, red arrows indicate the epithelial tongues, and the scale bar represents 1 mm (23). **(G)** Residual wound area on day 21 in human tumor necrosis factor knock-in, leptin receptor-deficient diabetic mice treated with phosphate-buffered saline (PBS), adalimumab (ADA), infliximab (IFX), golimumab (GOL), etanercept (ETN), certolizumab pegol (CZP), D2E7 F(ab′)2 [F(ab′)2 fragments of adalimumab], or D2E7-hCα1 [adalimumab containing an immunoglobulin A1 Fc domain] (*n* = 5 mice per group) (24). **(H)** Wound closure in streptozotocin-induced diabetic C57BL/6 mice assigned to the diabetic-wound control group (DW), FTO-knockout group (DW-koFTO), TRIB3-overexpression group (DW-oeTRIB3), or combined FTO-knockout and TRIB3-overexpression group (DW-koFTO-oeTRIB3) (*n* = 5 mice per group) (15). *, **, and *** indicate *p* < 0.05, *p* < 0.01, and *p* < 0.001, respectively, as reported in the original source studies.

In serial human acute wounds, single-cell and spatial profiling defined a temporal reference for keratinocyte migration and identified FOSL1 as a candidate transcriptional regulator of re-epithelialization (8). This reference enables comparison with DFU. DFU keratinocytes show early injury–response features but fail to differentiate. Liu et al. (7) distinguished keratinocyte abundance from the state by profiling normal skin, acute wound edges, and DFU edges. Analysis of ~100,000 cells from nine patients revealed thicker DFU epidermis with poorly differentiated keratinocytes, indicating increased abundance but impaired maturation and barrier restoration (Figure 1A). Basal cell 2 (BC-2) showed impaired basement-membrane continuity and reduced differentiation. Reduced BC-1/KRT15 expression was associated with poor basement-membrane continuity in DFU. BC-2 was enriched in cytokine-response and apoptosis-related programs, whereas the diabetes-associated keratinocyte (DAK) state was associated with apoptosis regulation and cellular glucose homeostasis. Together with the reduced differentiated keratinocyte compartments and lower KRT10 expression, these findings were consistent with impaired keratinocyte-state progression and epidermal maturation in DFU. Figure 1B schematically summarizes the inferred keratinocyte differentiation trajectories in acute wounds and DFUs.

Keratinocyte state composition correlates with clinical outcomes: enrichment of differentiated keratinocyte states is associated with healing. Theocharidis et al. (4) found that healing DFUs were enriched in differentiated keratinocytes and that LGALS7 was the top differentially expressed gene in keratinocytes from unwounded forearm skin of patients whose ulcers healed within 12 weeks. Its mechanistic and prognostic significance remains uncertain because the forearm analysis involved only a small subgroup.

Human single-cell data associate DFU epidermal failure with persistence of inflammatory basal-cell programs and reduced representation of differentiated keratinocyte states. These cells accumulate in the wound bed but lack the transcriptional programs required for stratification and barrier closure. The persistence of this dysfunction suggests that the epidermal arrest is maintained by dysregulated intercellular communication within the wound niche.

#### 2.1.2 Dysregulated epidermis–dermis signaling blocks differentiation

The pathological dermal microenvironment creates a regenerative block in the epidermis. Sustained inflammatory cytokines, excessive ECM degradation, and metabolic stress collectively suppress re-epithelialization. These factors prevent basal keratinocytes from carrying out migration and terminal differentiation programs. Consequently, barrier reconstruction fails despite the initiation of transcriptional stress responses.

Liu et al. (7) analyzed human normal skin, acute-wound edges, and DFU edges and identified increased CCL2^+^CXCL2^+^ fibroblasts and VWA1^+^ vascular endothelial cells in DFU (Figure 1C). CellChat analysis predicted communication from these populations, together with IL1B^+^ macrophages and GZMA^+^CD8^+^ T cells, toward BC-2 and DAK. Candidate signaling axes included IL1B–IL1R2, TNF–TNFRSF1A, IFNG–IFNGR1/2, and TNFSF12–TNFRSF12A; EGF and IGF were also among the inferred incoming pathways (Figure 1D). Laser-capture microdissection coupled with RNA sequencing further showed higher IL1R2 expression in DFU epidermis than in normal skin and acute wounds. SCENIC analysis showed reduced activity of differentiation-associated regulators, including IRF6, SIN3A, FOXC1, and EZH2, in BC-2 and increased activity of inflammation-associated regulators, including FOS and IRF3, in DAK. This regulatory pattern was consistent with impaired keratinocyte differentiation (7).

In outcome-stratified human DFU tissue, Theocharidis et al. (4) integrated spatial transcriptomics and immunostaining to map stromal niche programs. They identified a healing-associated fibroblast state defined by the enrichment of ECM-remodeling factors and inflammatory mediators, such as matrix metalloproteinase 1 (MMP1), MMP3, CHI3L1, and TNFAIP6. Spatially, these fibroblasts localize preferentially to the wound bed rather than the re-epithelializing wound edge. In healing ulcers, these fibroblasts form dense aggregates, whereas this organization is absent in non-healing tissues. This spatial arrangement suggests that restricted stromal niches provide necessary, localized remodeling cues. Conversely, the absence of such niches exposes the epidermis to diffuse, uncoordinated proteolytic and inflammatory signals. Choi et al. (6) validated healing-associated fibroblast programs in debrided human DFU tissue. CellChat analysis predicted recipient-specific signaling involving these cells: WNT2–FZD6 signaling linked healing-enriched fibroblasts with vascular endothelial cells, whereas IGF signaling linked them with basal keratinocytes and endothelial cells. In a preliminary race-stratified analysis, WNT signaling was more prominent in non-Hispanic Black patients, whereas IGF and Midkine signaling were stronger in White patients. Together with the spatial findings of Theocharidis et al., these results suggest that disruption of healing-associated stromal niches may reduce coordinated support for keratinocyte differentiation.

Transient inflammatory and hypoxic inputs can be converted into durable keratinocyte arrest via specific epigenetic and biochemical mechanisms. In the diabetic milieu, epigenetic constraints reinforce IL-1, TNF, and IFN-*γ* signaling. These modifications stabilize stress-responsive programs long after the initial stimulus has passed, effectively locking cells out of migratory and terminal differentiation states. In high-glucose human keratinocytes and diabetic mouse wounds, Yang et al. (14) showed that increased DNA hydroxymethylation and H3K4 trimethylation at the MMP9 promoter enhanced FOXO1-dependent MMP9 transcription. Elevated MMP9 impaired keratinocyte migration, whereas pharmacological inhibition of these epigenetic changes improved re-epithelialization and wound closure. At the epitranscriptomic level, Dong et al. (15) showed that FTO promoted keratinocyte proliferation and migration through TRIB3-mediated autophagy in high-glucose keratinocytes and streptozotocin-induced diabetic mice. This effect depended on N6-methyladenosine (m6A)-YTHDF2-mediated regulation of TRIB3 mRNA stability. Furthermore, chromatin accessibility directly constrains migration. In murine wound-edge keratinocytes, Kashgari et al. (16) used an assay for transposase-accessible chromatin with high-throughput sequencing to show that wound-induced chromatin opening at the Fscn1 enhancer depends on GRHL3. This enhancer activation fails in diabetic wounds, linking perturbed chromatin dynamics to stalled collective migration. Finally, biochemical modifications can render abundant signals functionally inert. Oncostatin M normally promotes re-epithelialization via signal transducer and activator of transcription 3 and TP63-ITGB1 induction. Across human wound fluid, cultured human keratinocytes, and db/db mouse wounds, Das et al. (17) examined the effect of glycation on oncostatin M activity. Glycation reduced its pro-re-epithelialization activity, whereas deglycation restored activity in the experimental systems studied. These convergent mechanisms frame epidermal arrest as a reinforced regulatory state, wherein inflammatory inputs persist while migration- and differentiation-enabling programs remain constrained. Taken together, high glucose solidifies transient inflammatory inputs into persistent stress programs, chronically suppressing migration and differentiation.

In summary, epidermal arrest results from the convergence of extrinsic inflammatory and proteolytic constraints. Sustained cytokine signaling and hypoxic stress within the dermal niche prevent keratinocytes from executing differentiation programs. Crucially, metabolic memory mechanisms spanning epigenetic, transcriptomic, and biochemical layers transform transient environmental stimuli into persistent intracellular stress states. This consolidation renders the epidermis refractory to repair despite the absence of the initial trigger.

#### 2.1.3 Blockade in epidermal lineage progression traps differentiation

Trajectory inference localizes the primary DFU defect to a blockade in early lineage progression. Pathologically, the epidermis fails to make the critical transition from inflammatory activation to proliferation and migration. This arrest leads to the accumulation of stress-responsive basal progenitors and the depletion of transient migratory intermediates. As a result, the lineage remains trapped in a non-restorative cycle that precludes terminal differentiation and barrier reconstruction.

To define this blockade at cellular resolution, Liu et al. (7) applied pseudotime trajectory inference to reconstruct keratinocyte differentiation lineages across normal skin, acute wounds, and DFU. The DFU trajectory shows pathological accumulation in stress- and diabetes-associated basal states (Figure 1E). In particular, basal cells accumulate in a BC-2 state enriched for TXNIP, IL1R2, and interferon-stimulated genes, alongside a distinct DAK-associated transcriptional state. This basal block coincides with depletion of late differentiation compartments and reduced KRT10 protein expression.

Longitudinal profiling further pinpoints this divergence to the failure of an early transition from inflammation to proliferation. In serial human acute wounds sampled on days 1, 7, and 30, Li et al. (18) identified SNHG26 as a transient regulator of the inflammatory-to-proliferative transition. They identified the lncRNA SNHG26 as a transient regulator induced in basal keratinocytes during the inflammatory and proliferative phases. In DFU, reduced SNHG26 expression indicates a specific failure to activate this inflammation-to-proliferation module. In addition, across serial human acute-wound samples and an external human DFU bulk-transcriptomic dataset, Zhang et al. (19) associated HOXC13-AS with epidermal differentiation. Its expression is typically downregulated early after injury to prioritize migration but fails to recover in chronic wounds, blocking re-entry into terminal differentiation. Together, these datasets show that epidermal arrest results from an inability to complete the early transition from inflammation to proliferation, which subsequently prevents re-entry into terminal differentiation.

In normal human keratinocytes and murine acute wounds, Siriwach et al. (20) identified a THBS1^+^ transitional population that emerged from basal keratinocytes, moved toward the wound front, and subsequently entered terminal differentiation. This study did not assess DFU tissue. Human DFU single-cell data show accumulation of stress-associated basal states together with reduced differentiated keratinocyte compartments (7). In an outcome-stratified human cohort, differentiated keratinocytes were enriched in healing DFUs relative to non-healing DFUs (4). Together, these findings are consistent with impaired productive lineage progression in DFU, but they do not directly demonstrate depletion of the THBS1^+^ transitional population.

In DFU, keratinocytes lose intrinsic lineage plasticity and become trapped in a self-reinforcing inflammatory basal state. This lineage arrest actively prevents terminal differentiation, leaving the epidermis unresponsive to generalized regenerative cues. Accordingly, potential Axis I interventions may be better conceptualized as state-directed approaches that encourage pathological basal-cell programs to re-enter migratory and differentiation trajectories while preserving the healthy basal/progenitor continuum required for epidermal regeneration (7, 21, 22).

#### 2.1.4 Targeting inflammation to restore keratinocyte regeneration

Axis I-directed therapies are better framed as approaches that may support keratinocyte-lineage progression. Attenuating chronic inflammatory-niche signals may help reduce the persistence of pathological basal-cell states, provided that the basal/progenitor continuum required for normal epidermal regeneration is preserved. Within this framework, two complementary directions are supported: (1) spatiotemporally limited modulation of receptor–ligand signaling to reduce upstream inflammatory inputs, (2) epigenetic or epitranscriptomic modulation to mitigate hyperglycemia-associated molecular memory and support recovery of migration and differentiation competence.

First, modulating upstream inflammatory ligands may attenuate signals that sustain arrest-associated keratinocyte programs. In Lepr^db/db^ wounds, Tan et al. (23) fused IL-1Ra to the ECM-binding PlGF_123–141_ peptide, prolonging its retention at the wound site and sustaining local IL-1R1 blockade. Genetic loss of Il1r1 accelerated wound closure, with near-complete re-epithelialization by day 9. A single topical dose of matrix-binding IL-1Ra improved re-epithelialization and wound closure (Figure 1F) and reduced pro-inflammatory cytokines and MMPs in the wound. In a human TNF knock-in db/db mouse model, Cao et al. (24) found that TNF inhibitors differed in their effects on cutaneous wound healing. At day 21, adalimumab and infliximab produced greater reductions in residual wound area than etanercept or certolizumab pegol (Figure 1G). Given the microbial burden of DFUs, systemic TNF inhibition requires careful safety evaluation. These preclinical findings support further evaluation of locally retained or time-limited anti-inflammatory strategies.

Second, epigenetic and epitranscriptomic modulation may help restore the temporal coordination of keratinocyte migration and differentiation. Spatiotemporal mapping indicates that keratinocyte migration and terminal differentiation are temporally coordinated during repair. In high-glucose human keratinocytes and diabetic mouse wounds, Yang et al. (14) showed that inhibition of DNA demethylation and histone H3 methylation rescues diabetic re-epithelialization. Mechanistically, high glucose drives FOXO1 binding at the MMP9 promoter. The resulting MMP9 overexpression suppresses migration, a defect reversible by pharmacologic epigenetic blockade. In addition, RNA modifications appear to contribute to repair competence. In keratinocyte and diabetic-mouse wound experiments, Hu et al. (25) identified a lactylation-regulated METTL3–m6A–HNRNPA2B1 pathway associated with keratinocyte proliferation and wound repair. Expanding this landscape, Dong et al. (15) revealed that the m6A demethylase FTO functions as a glucose-sensitive rheostat. Hyperglycemia suppresses FTO expression, triggering YTHDF2-mediated decay of TRIB3 mRNA. This blockade inhibits autophagy-dependent migration, whereas restoring the FTO-TRIB3 axis accelerates diabetic wound closure, significantly reducing unhealed wound areas (Figure 1H).

Collectively, these strategies may relieve constraints on keratinocyte-lineage progression by reducing inhibitory inflammatory inputs and mitigating hyperglycemia-associated epigenetic or epitranscriptomic memory. Axis I-directed intervention should therefore be interpreted as state-directed modulation or reprogramming of pathological basal-cell programs rather than as a strategy for removing defined basal-cell subsets. Its therapeutic effect may remain limited if upstream immune-stromal drivers in Axis II continue to supply inflammatory and proteolytic signals, and any newly formed barrier may remain vulnerable to regression. Importantly, current evidence does not directly demonstrate that these interventions can selectively modulate pathological basal-cell states without affecting healthy epidermal progenitors. Future validation should combine single-cell pharmacodynamic profiling, spatial mapping, and functional endpoints to assess preservation of KRT5/KRT14-positive basal cells, proliferative basal cells, migratory intermediates, and terminal differentiation programs (26–28).

### 2.2 Axis II—the immune-stromal trap

In DFU, persistent non-healing is partly driven by combined immune activation and stromal dysfunction. Single-cell and spatial profiling reveal enrichment of inflammatory myeloid states and cytotoxic T-cell and NK-cell populations, together with loss of repair-associated fibroblast subsets. Chemokine networks sustain leukocyte recruitment, while neutrophil extracellular trap (NET)-associated proteases and increased matrix metalloproteinase activity promote ECM degradation and generate pro-inflammatory matrix fragments. Together, these processes sustain inflammation and impair productive matrix remodeling. Current therapeutic approaches under investigation include stage-matched modulation of leukocyte recruitment and proteolysis, promotion of efferocytosis and inflammatory resolution, and stabilization and rebuilding of the ECM in protease-rich wounds.

#### 2.2.1 Immune and fibroblast heterogeneity

Single-cell profiling distinguishes DFU inflammation into distinct immune activation states and fibroblast subpopulations, detailing the transition from inflammatory phases to ECM reconstitution. Physiological repair requires a coordinated shift in macrophage and fibroblast programs from inflammation to matrix restoration. In contrast, non-healing DFUs display persistent inflammatory phenotypes and a specific loss of reparative, matrix-associated fibroblast lineages.

**Figure 2:**
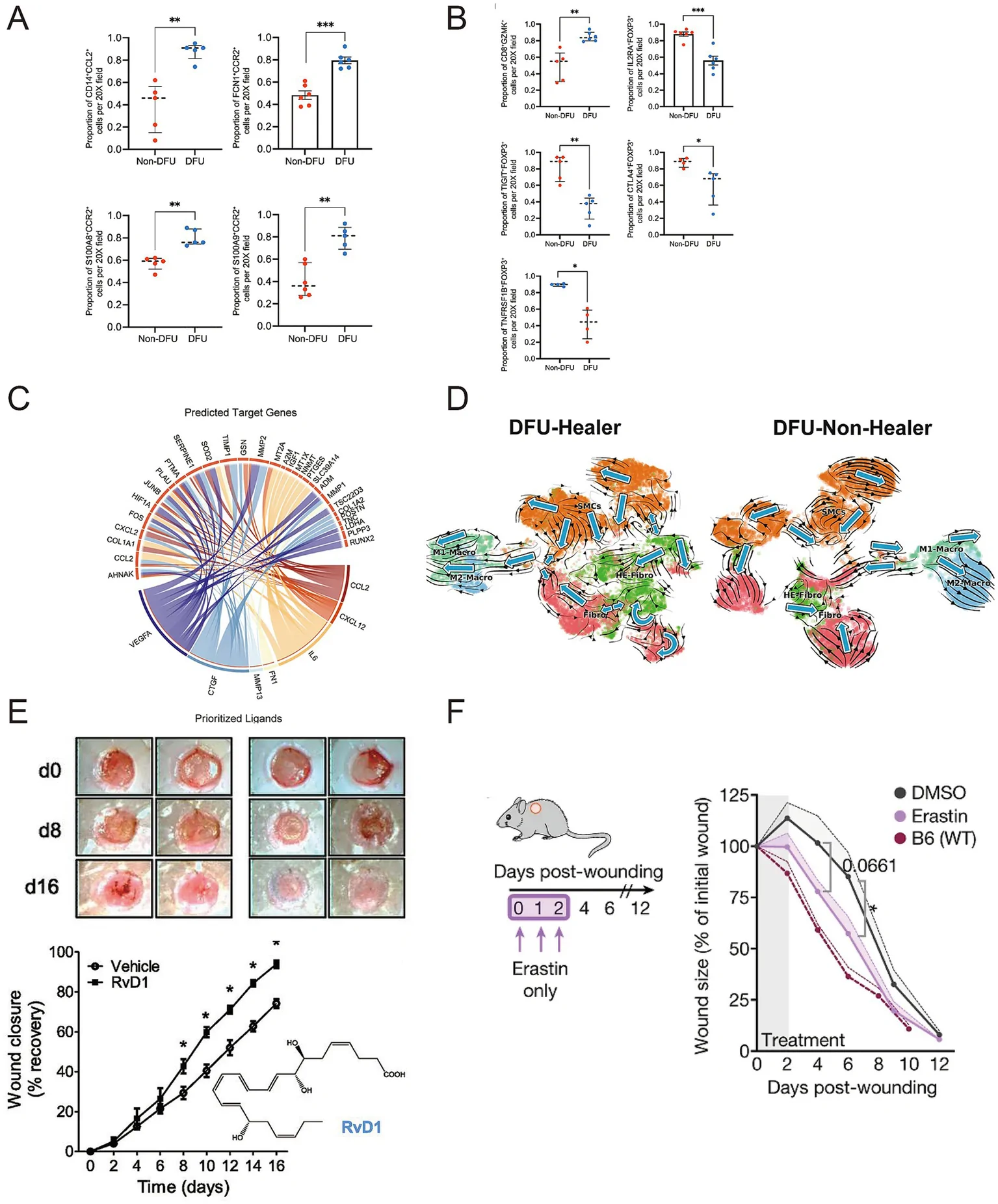
Immune–stromal dysregulation and preclinical modulation of inflammatory resolution in diabetic wounds. **(A)** Quantitative immunofluorescence analysis of CD14^+^CCL2^+^ monocyte population 1 cells and FCN1^+^CCR2^+^, CCR2^+^S100A8^+^, and CCR2^+^S100A9^+^ monocyte population 2 cells in human granulation tissue from diabetic foot ulcers and non-diabetic traumatic wounds (*n* = 5–6 patients per group) (29). **(B)** Quantitative immunofluorescence analysis of CD8^+^GZMK^+^ effector T cells and IL2RA^+^FOXP3^+^, TIGIT^+^FOXP3^+^, CTLA4^+^FOXP3^+^, and TNFRSF1B^+^FOXP3^+^ regulatory T-cell populations in human granulation tissue from diabetic foot ulcers and non-diabetic traumatic wounds (*n* = 4–6 patients per group) (29). **(C)** NicheNet-predicted ligand–target network derived from human diabetic foot ulcer single-cell data. Healing-enriched fibroblast subcluster 3 represents the sender population, with its ligands shown in the lower semicircle; subclusters 4, 6, and 13 represent the receiver populations, with their predicted target genes shown in the upper semicircle (4). **(D)** RNA velocity trajectories among fibroblasts, healing-enriched fibroblasts, smooth muscle cells, and M1 and M2 macrophage states in human healing diabetic foot ulcers (*n* = 7 patients) and non-healing diabetic foot ulcers (*n* = 4 patients). Black streamlines show local velocity vectors, and blue arrows show the overall velocity direction (4). **(E)** Representative wounds and wound-closure kinetics in splinted full-thickness dorsal wounds of leptin receptor-deficient diabetic mice treated with vehicle or resolvin D1 (*n* = 5–9 per group) (38). **(F)** Wound-closure kinetics in leptin receptor-deficient diabetic mice treated with dimethyl sulfoxide vehicle (*n* = 7) or erastin alone (*n* = 8), with normoglycemic C57BL/6 wild-type mice shown as the reference group (*n* = 8) (39). *, **, and *** indicate *p* < 0.05, *p* < 0.01, and *p* < 0.001, respectively, as reported in the original source studies.

Regarding immune heterogeneity, Gan et al. (29) identified two monocyte populations enriched in human DFU granulation tissue. CD14^+^CCL2^+^ Mono1 cells expressed inflammatory cytokines and chemokines, whereas FCN1^+^CCR2^+^ Mono2 cells showed blood-derived and extravasation-associated features. Immunofluorescence confirmed increased proportions of CD14^+^CCL2^+^, FCN1^+^CCR2^+^, CCR2^+^S100A8^+^, and CCR2^+^S100A9^+^ cells in DFU (Figure 2A). The same study found increased CD8^+^GZMK^+^ effector T cells and reduced IL2RA^+^FOXP3^+^, TIGIT^+^FOXP3^+^, CTLA4^+^FOXP3^+^, and TNFRSF1B^+^FOXP3^+^ regulatory T-cell populations in DFU (Figure 2B). In an outcome-stratified human DFU cohort, Theocharidis et al. (4) observed enrichment of IL1B/S100A8/S100A9-high inflammatory myeloid states in healing DFUs, whereas non-healing DFUs contained more C1QA/B/C-high macrophage and NKG7/GNLY-high cytotoxic populations. In addition, across human diabetic neutrophils and PAD4-deficient diabetic mice, Wong et al. (30) demonstrated that diabetes upregulates peptidyl arginine deiminase 4 (PAD4), shifting neutrophil programs toward the formation of neutrophil extracellular traps (NETs).

Across human DFU single-cell cohorts, stromal profiling revealed fibroblast programs associated with clinical outcome. In healing DFUs, healing-enriched fibroblast subclusters expressed inflammatory and matrix-remodeling genes, including IL6, CHI3L1, MMP1, MMP3, ASPN, and POSTN, and related programs were confirmed in debrided ulcers (4, 6). DFU tissue also showed expansion of CCL2^+^CXCL2^+^ fibroblasts together with increased GZMA^+^CD8^+^ T cells (7). Together, reduced representation of healing-associated fibroblast programs and expansion of chemokine-active stroma provide a cellular context for the recruitment and matrix-degradation cycle described later.

#### 2.2.2 Chemokine recruitment and ECM breakdown

Chemokine-driven leukocyte recruitment and protease-mediated ECM degradation maintain the chronic inflammatory microenvironment. Theocharidis et al. (4) used NicheNet analysis of outcome-stratified human DFU tissue to predict ligand–target relationships among healing-enriched fibroblast subclusters. Healing-enriched fibroblast subcluster 3 was defined as the sender population, and its prioritized ligands were linked to predicted target genes in receiver subclusters 4, 6, and 13 (Figure 2C). Gan et al. (29) identified complementary CCL2- and CCR2-expressing myeloid populations in human DFU granulation tissue. CD14^+^CCL2^+^ Mono1 cells and CCL2-expressing Macro1 cells were the inferred ligand sources, while FCN1^+^CCR2^+^ Mono2 cells formed the receptor-expressing, blood-derived population. CellPhoneDB predicted stronger CCL2–CCR2 communication from Mono1/Macro1 to Mono2 in DFU.

Protease dysregulation represents the second component of this cycle. In a human DFU wound-fluid cohort, Liu et al. reported that the MMP-9/TIMP-1 ratio is elevated and TGF-β1 is reduced in the wound fluid of non-healing ulcers. A combined biomarker panel predicted healing outcomes with high accuracy (area under the curve: 0.94) (31). Neutrophils further amplify proteolysis through NET formation. In human diabetic neutrophils and diabetic mouse wounds, Wong et al. showed that diabetes primes neutrophils for PAD4-dependent neutrophil extracellular trap formation (NETosis). These chromatin structures concentrate neutrophil elastase and MMP-9 to degrade the local matrix. Genetic PAD4 deletion accelerates diabetic wound closure (30).

ECM fragmentation adds a further inflammatory input. Hyaluronan fragments engage toll-like receptor 2 to induce pro-inflammatory gene expression, while collagen- and elastin-derived matrikines can act as chemotactic or immunomodulatory signals (32, 33). These mechanisms provide plausible explanations for how excessive ECM fragmentation may reinforce inflammation in DFU, but they have not been directly demonstrated as a complete pathway in human DFU tissue. Theocharidis et al. (4) distinguished healing-associated remodeling marked by MMP1, MMP3, and MMP11 from protease-biased matrix destruction in non-healing DFUs. Within this environment, chemokine gradients increase leukocyte entry, infiltrating cells intensify proteolytic and oxidative injury, and newly generated matrix fragments further sustain inflammatory signaling. The interaction between recruitment and protease-biased turnover prevents coordinated resolution and productive matrix remodeling, completing the immune-stromal trap.

#### 2.2.3 Failed transition from inflammation to remodeling

Human studies consistently link failure of inflammatory resolution to loss of reparative stromal programs, but they do not reveal which compartment changes first. Theocharidis et al. (4) inferred stronger connectivity among fibroblast, healing-enriched fibroblast, smooth muscle cell, and macrophage states in healing DFUs, whereas non-healing DFUs showed more separated trajectories and weaker predicted state bridges (Figure 2D). Choi et al. (6) independently confirmed enrichment of healing-associated fibroblast programs in debrided ulcers that healed. In a separate DFU-versus-non-DFU granulation-tissue cohort, Gan et al. (29) observed expansion of inflammatory monocyte populations together with loss of COL6A1^+^COL6A3^+^ and COL7A1^+^COL10A1^+^ fibroblast subsets. These findings support reciprocal immune–stromal dysregulation without establishing temporal order. Myeloid inflammation may suppress fibroblast repair, fibroblast-derived chemokines may sustain immune recruitment, and shared metabolic, ischemic, microbial, or matrix injury may reinforce both programs.

Perturbation studies provide directional information, although the two arms have not been tested in equivalent disease contexts. In macrophage–fibroblast co-culture and diabetic mouse-wound experiments, diabetic macrophages suppressed fibroblast expression of MMP1, MMP3, MMP11, CHI3L1, and VEGFA; non-diabetic macrophages or IL6 supplementation restored elements of this program, and macrophage-specific IL6 improved wound healing and angiogenesis in diabetic mice (34). In acute mouse wounds, fibroblast-specific Ccl2 deletion reduced early CCR2^+^ monocyte/macrophage recruitment in acute wounds, demonstrating that fibroblast-derived CCL2 contributes to physiological myeloid entry after injury (35). The first model directly tests myeloid-to-fibroblast regulation under diabetic conditions, whereas the second demonstrates stromal control of acute recruitment without showing that intrinsic fibroblast defects drive chronic immune accumulation in human DFUs. A universal initiating compartment therefore cannot be assigned. Once chronicity is established, persistent myeloid inflammation, impaired fibroblast repair, and proteolytically damaged ECM may instead maintain one another, and the process that initiated the wound may no longer be the process that sustains non-healing.

#### 2.2.4 Breaking the immune-stromal inflammatory loop

The distinction between initiation and maintenance changes the therapeutic question. Axis II treatment should be matched to the dominant reversible process in the current wound: excessive recruitment and protease-mediated injury call for control of destructive myeloid activity, failed resolution or efferocytosis calls for pro-resolution reprogramming, and impaired fibroblast repair or matrix instability requires restoration of a repair-permissive stromal environment. When several processes sustain one another, sequential or combined intervention may be needed to achieve a durable transition toward resolution and remodeling.

Inflammatory dominance does not imply that all myeloid recruitment should be suppressed. In human diabetic-wound samples, Cunnion et al. (36) detected elevated C5a and C3 fragments. In complementary diabetic-mouse intervention experiments, classical complement-pathway inhibition with peptide inhibitor of complement C1 reduced leukocyte infiltration. Sawaya et al. (37) identified a different defect: TREM1-FOXM1 signaling was attenuated in non-healing DFUs, and topical TREM1 activation in db/db mice increased FOXM1^+^ neutrophil recruitment while reducing citH3^+^ NET formation and improving healing kinetics. These interventions separate leukocyte burden from leukocyte state. Treatment may need to limit destructive recruitment and proteolysis while preserving or restoring myeloid programs that support acute repair.

Where defective clearance and persistent inflammatory signaling maintain the loop, pro-resolution treatment may facilitate the transition toward repair. In db/db mice, topical resolvin D1 accelerated wound closure (Figure 2E). The same study also showed enhanced macrophage phagocytosis and reduced accumulation of apoptotic cells in diabetic wounds (38). In mouse dendritic-cell assays, Maschalidi et al. (39) found that genetic loss or pharmacological inhibition of SLC7A11 increased efferocytosis. In db/db mice, erastin alone accelerated wound closure (Figure 2F). In a separate regimen combining apoptotic-cell administration with erastin, SLC7A11 inhibition reduced apoptotic-cell burden in diabetic wounds. In diabetic mouse wounds, topical mineralocorticoid-receptor blockade shifted macrophages toward an M2-associated phenotype and increased macrophage expression of angiogenic factors (40). These approaches promote inflammatory termination and downstream repair without assuming that reduced immune-cell abundance alone will restore the wound.

Matrix-directed treatment becomes relevant once proteolytic turnover has destabilized the stromal scaffold. In a randomized trial, collagen/oxidized regenerated cellulose dressing produced a numerically higher 12-week closure rate than control, but the overall difference was not statistically significant; a *post hoc* subgroup with ulcers of less than six months showed only borderline benefit (41). A subsequent pilot study found that the same matrix lowered the MMP-9/TIMP-2 ratio and was accompanied by greater wound-area reduction over eight days, although wound-area change was not its primary endpoint (42). Stronger clinical evidence comes from the Explorer trial, in which a sucrose octasulfate dressing increased 20-week closure from 30 to 48% in neuroischemic DFUs receiving standard care (43). The variable strength of these findings makes wound duration, ischemic burden, and residual stromal plasticity important when positioning matrix therapy within Axis II.

Durable interruption of Axis II may therefore require stage-matched combinations that limit persistent recruitment of inflammatory monocytes and neutrophils, reduce excessive proteolysis, promote efferocytic clearance and inflammatory resolution, and support repair-associated fibroblast and ECM programs. Even after the immune–stromal loop is attenuated, tissue viability remains dependent on adequate perfusion and neural regulation; persistent deficits in these support systems are addressed in Axis III.

### 2.3 Axis III—vascular dysfunction and neurovascular uncoupling

Microvascular dysfunction in DFUs is marked by inflammatory and stress-related endothelial states with reduced angiogenic capacity, contributing to hypoperfusion, persistent hypoxia, and impaired tissue support. Neural dysfunction and incomplete reinnervation occur in the same microenvironment. Eccrine glands may remain morphologically identifiable while losing sudomotor activity and peri-glandular neural and vascular support; this pattern represents an adnexal manifestation of broader neurovascular injury and may locally amplify barrier vulnerability, rather than accounting for deep ischemia or stromal arrest on its own. Persistent endothelial hyporesponsiveness can coexist with vascular rarefaction and basement-membrane remodeling. Therapeutic strategies therefore span perfusion management, endothelial reactivation, coordinated vessel–nerve repair, recovery of adnexal function, and mechanical offloading to protect regenerating tissue.

#### 2.3.1 Endothelial stress states and impaired angiogenesis

Single-cell atlases attribute microvascular dysfunction primarily to altered endothelial cell states. Physiological repair requires a transition from quiescent to angiogenic phenotypes. However, DFU endothelium occupies stress-associated and inflammatory states that lack angiogenic ability. Consequently, even when capillary density appears histologically preserved, this qualitative shift prevents the formation of a robust, perfusion-competent network.

**Figure 3:**
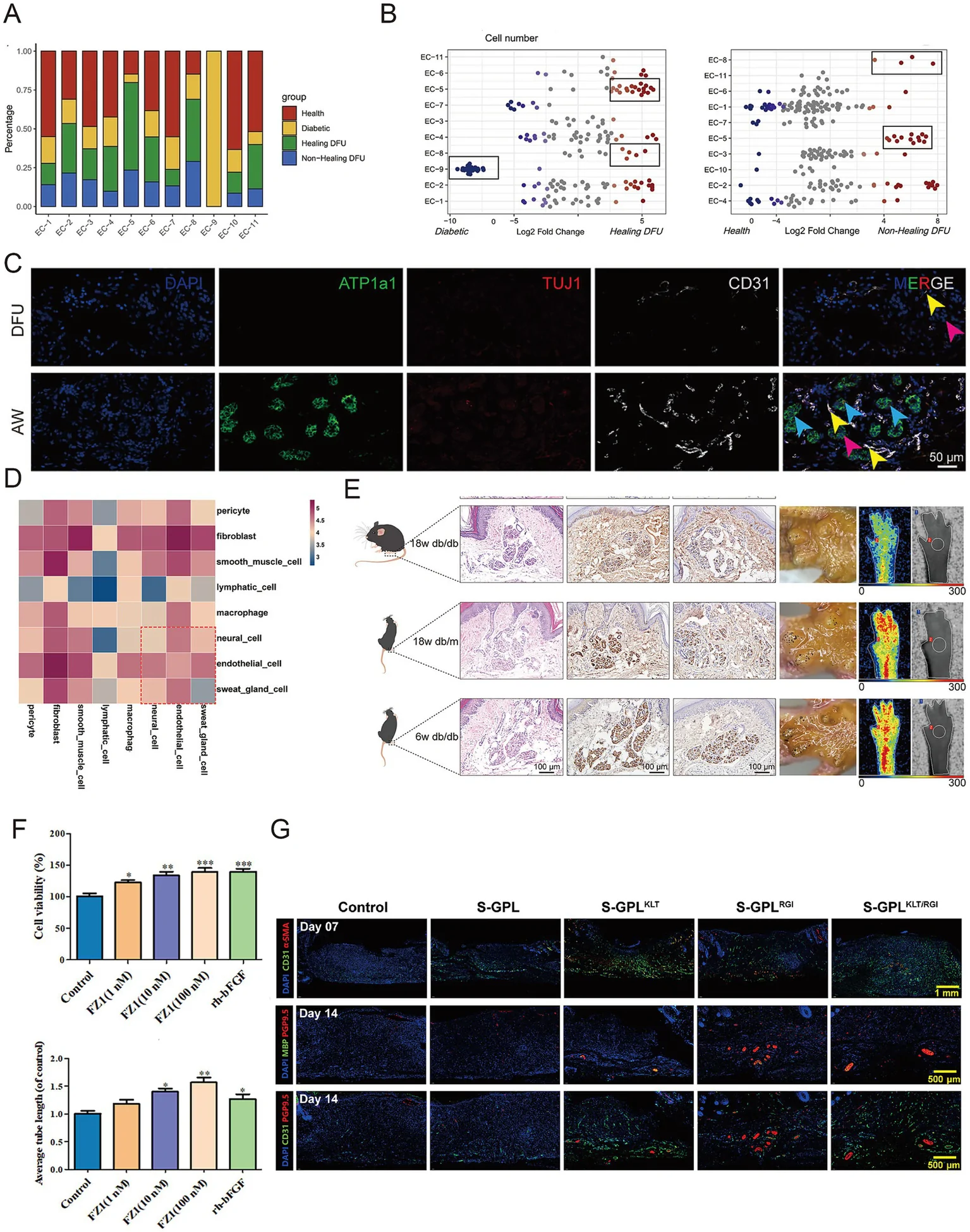
Endothelial heterogeneity, neurovascular–eccrine dysfunction, and experimental restoration in diabetic wounds. **(A)** Relative proportions of endothelial-cell subclusters EC-1 to EC-11 in a computational re-analysis of human foot-skin single-cell RNA-sequencing data from healthy individuals without diabetes (*n* = 11), individuals with diabetes without foot ulcers (*n* = 8), patients with healing diabetic foot ulcers (*n* = 9), and patients with non-healing diabetic foot ulcers (*n* = 5) (44). **(B)** Differential abundance of endothelial-cell subclusters, expressed as log_2_ fold change, across comparisons between diabetic skin and healing diabetic foot ulcers and between healthy skin and non-healing diabetic foot ulcers. Red and blue points indicate enrichment toward the group shown on the right and left of each comparison, respectively (44). **(C)** Representative immunofluorescence images of sweat glands in human diabetic foot ulcer-edge and acute-wound-edge tissues stained for ATPase Na^+^/K^+^-transporting subunit alpha 1, class III beta-tubulin, and platelet endothelial cell adhesion molecule 1, indicating sweat-gland function, innervation, and vasculature, respectively. Scale bar, 50 μm (46). **(D)** Heatmap of CellPhoneDB-inferred interactions among dermal cell populations in normal human skin, highlighting communication involving neural cells, endothelial cells, and sweat-gland cells. Color intensity represents the inferred interaction strength (46). **(E)** Morphological and functional assessment of sweat glands in an unwounded paw model using 18-week-old leptin receptor-deficient diabetic mice, age-matched heterozygous control mice, and 6-week-old leptin receptor-deficient diabetic mice (*n* = 3 per group). The panel includes hematoxylin-and-eosin staining, immunohistochemical staining for ATPase Na^+^/K^+^-transporting subunit alpha 1 and cytokeratin 18, sweat-dot assays, and laser Doppler paw-perfusion maps. Region of interest 1 represents the entire paw pad, and region of interest 2 represents the sweat-gland region. The perfusion color scale ranges from low flow in blue to high flow in red. Scale bar, 100 μm (46). **(F)** Proliferative activity and average capillary-like tube length of human umbilical vein endothelial cells treated with vehicle control, FZ1 at 1, 10, or 100 nM, or recombinant human basic fibroblast growth factor at 100 ng/mL (*n* = 3 per condition) (56). **(G)** Representative immunofluorescence images of streptozotocin-induced diabetic rat wounds treated with saline; a sprayable gelatin methacryloyl–methacrylamide-modified *ε*-poly-L-lysine hydrogel sponge; the hydrogel containing the vascular endothelial growth factor-mimetic peptide KLT; the hydrogel containing the brain-derived neurotrophic factor-mimetic peptide RGI; or the hydrogel containing both peptides (*n* = 3 per group). Platelet endothelial cell adhesion molecule 1 and alpha-smooth muscle actin staining on day 7 show neovascularization and vessel maturation. Myelin basic protein and protein gene product 9.5 staining on day 14 show nerve regeneration, and dual platelet endothelial cell adhesion molecule 1/protein gene product 9.5 staining shows the spatial association between vessels and nerve fibers. Scale bars: 1 mm for day 7 and 500 μm for day 14 (57). *, **, and *** indicate *p* < 0.05, *p* < 0.01, and *p* < 0.001, respectively, as reported in the original source studies.

In a computational re-analysis of human DFU endothelial-cell data, Zhao et al. (44) resolved this heterogeneity into 11 distinct endothelial subsets (Figure 3A). Their analysis distinguished healing from non-healing states based on relative occupancy. Non-healing ulcers are enriched for the EC-5 subset, which is characterized by acute inflammatory programs and persistent TNF signaling. Conversely, the healing-enriched EC-8 subset couples stress adaptation to metabolic reprogramming via Adenosine 5′-monophosphate-activated protein kinase and peroxisome proliferator-activated receptor signaling (Figure 3B). EC-8 also shows stronger oxidative phosphorylation and hypoxia signatures. In addition, specific subsets express epithelial-mesenchymal transition markers, favoring permeability over barrier function.

In a computational re-analysis of the human DFU dataset GSE165816, Zhang et al. (45) identified an ENTPD1^+^ endothelial state enriched in healing samples. Trajectory and pathway analyses associated this state with purinergic metabolism and reduced ACKR1–MIF signaling. These findings suggest that angiogenic stimulation alone may be insufficient when inflammatory endothelial programs persist. These endothelial dysfunctions do not occur in isolation. Single-cell maps show that the endothelium is functionally linked to skin appendages, forming a coordinated network that deteriorates under hypoxic conditions.

#### 2.3.2 Hypoxia-associated neurovascular and eccrine dysfunction

The endothelial abnormalities described earlier extend to the peri-adnexal microenvironment, where eccrine secretory function depends on local vascular and neural support. In human DFU tissue, Guo et al. (46) observed morphologically preserved sweat glands with reduced ATP1A1 expression and diminished periglandular CD31-positive vascular and class III β-tubulin-positive neural signals (Figure 3C). These findings document a structural-functional dissociation and the spatial co-occurrence of glandular, vascular, and neural abnormalities. Their cross-sectional design does not resolve the temporal sequence or causal relationships among these changes.

Hyperglycemia can also impair hypoxic adaptation in compartments not directly related to sweat-gland function. In human dermal fibroblasts and microvascular endothelial cells, hyperglycemia impaired HIF-1α stabilization and attenuated hypoxia-responsive angiogenic signaling, providing a plausible mechanism for broader vascular and stromal dysfunction in diabetic tissue (47). Luo et al. (48) separately found reduced sweat-gland innervation in patients with type 2 diabetes. The innervation index was associated with glycated hemoglobin and was lower in feet with anhidrosis, placing sudomotor denervation within the broader context of diabetic neuropathy.

CellPhoneDB analysis of normal human skin predicted candidate ligand–receptor interactions among neural, endothelial, and sweat-gland cells (Figure 3D). These inferred relationships provide a reference framework for interpreting the spatially coordinated neural, vascular, and eccrine abnormalities observed in DFU tissue; they do not directly demonstrate loss of communication in DFU. In complementary *in vitro* co-culture experiments, neural cells increased sweat-gland functional markers, whereas sweat-gland cells promoted neural-cell migration, proliferation, and neurite outgrowth, supporting the possibility of reciprocal neuro-eccrine signaling under defined culture conditions (46).

Reduced sweating may promote xerosis and fissuring, thereby weakening barrier integrity and increasing susceptibility to repetitive injury and secondary infection (46). Eccrine dysfunction may therefore contribute to wound-edge vulnerability, although its independent effect on ulcer closure has not been quantified. Restoring sudomotor innervation may improve eccrine function and local barrier support, but available evidence does not indicate that it would, by itself, correct diffuse ischemia, endothelial arrest, basement-membrane remodeling, or deep stromal repair failure. These broader abnormalities involve interacting vascular, neural, metabolic, and matrix compartments. Their chronic remodeling is considered in the next section.

#### 2.3.3 Restricted endothelial plasticity and chronic neurovascular remodeling

Vascular repair requires endothelial cells to enter transient proliferative and angiogenic programs before vessel maturation. In human non-healing DFUs, Lu et al. (49) reported downregulation of CCND1, ENO1, HIF-1α, and SERPINE1, together with trajectory-inferred restriction of endothelial state transitions. This profile is consistent with limited proliferative and metabolic responsiveness to reparative cues. Pseudotime captures transcriptional relationships among cell states and supports reduced state plasticity without reconstructing the temporal progression from acute injury to chronic vascular damage.

Evidence from animal models and human tissue places this endothelial restriction within broader neurovascular and matrix remodeling. In an unwounded db/db paw model, Guo et al. (46) found that 18-week diabetic mice showed more severe sudomotor dysfunction, lower perfusion, and greater periglandular neurovascular abnormalities than 6-week diabetic mice (Figure 3E), consistent with progressive disruption of the local neurovascular environment during diabetes. In amputated diabetic limbs, Sugiyama et al. (50) reported type IV collagen-rich basement-membrane thickening around eccrine structures, documenting peri-adnexal matrix alteration in advanced diabetic tissue. Galkowska et al. (51) also detected growth-associated protein 43 expression in DFU tissue despite reduced protein gene product 9.5-positive nerve fibers, a pattern consistent with regenerative signaling that does not culminate in restoration of the local neural network. These observations indicate that chronic diabetic tissue remodeling is not confined to endothelial transcriptional states but also involves neural and peri-adnexal matrix compartments.

In chronic DFUs, restricted endothelial plasticity may coexist with incomplete neural regeneration and peri-adnexal matrix remodeling, creating multiple constraints on neurovascular recovery. Current studies span different species, sampling designs, and readouts and do not reconstruct the full temporal evolution of human DFUs. They neither identify a single initiating lesion nor establish that these changes are irreversible. Peri-eccrine basement-membrane thickening documents local remodeling but does not measure the extent of deep stromal arrest across DFU tissue. These abnormalities nevertheless provide complementary therapeutic entry points at the levels of perfusion, endothelial responsiveness, neural support, and matrix remodeling.

#### 2.3.4 Restoring neurovascular function and protecting the niche

Treatment of Axis III requires a hierarchy that distinguishes foundational clinical management from context-specific adjuncts and emerging mechanism-directed strategies. Debridement, infection control, glycemic management, offloading, and assessment and, where indicated, restoration of limb perfusion form the clinical foundation. Residual microvascular, endothelial, or neural dysfunction may require concurrent interventions selected according to the dominant reversible constraint within each wound. Within this hierarchy, eccrine recovery represents a potential contributor to local sudomotor function and barrier maintenance.

Clinical studies support oxygen therapy as a context-dependent adjunct. In the Hyperbaric Oxygen Therapy in Diabetics with Chronic Foot Ulcers trial, hyperbaric oxygen improved healing in selected patients with chronic DFUs when added to multidisciplinary care (52). In the cyclical topical wound oxygen trial, cyclical topical oxygen improved 12-week closure in ulcers that met prespecified perfusion criteria and received debridement, offloading, and standard wound care (53). In a before-and-after study of patients undergoing lower-extremity revascularization, Arora et al. (54) found increased cutaneous microcirculatory perfusion, whereas neurogenic axon-reflex vasodilation remained impaired. These interventions therefore occupy distinct positions in the treatment hierarchy. Vascular assessment and revascularization address arterial inflow, oxygen therapy provides selective adjunctive support, and residual neurogenic microvascular dysfunction may require additional treatment.

Preclinical studies identify endothelial responsiveness as an additional and potentially reversible constraint. In diabetic ischemic mice, tissue nanotransfection-mediated demethylation of the endothelial PLCγ2 promoter restored VEGF responsiveness, improved tissue perfusion, and accelerated wound closure (55). In HUVECs, the integrin *α*vβ3 agonist FZ1 increased endothelial proliferative activity and capillary-like tube formation (Figure 3F). Mechanistic experiments linked these effects to direct αvβ3 engagement, FAK/ERK/AKT activation, and VEGFC induction; in diabetic mice, FZ1 increased CD31 and α-SMA expression and accelerated wound closure (56). In diabetic mice, SH3BGRL3 overexpression likewise promoted angiogenesis and wound closure in diabetic models (44). Across these models, therapeutic benefit was associated with restored endothelial signaling and angiogenic competence, positioning endothelial hyporesponsiveness as a candidate treatment node within Axis III. Clinical relevance remains to be established, particularly in advanced DFUs with limited tissue viability.

Coordinated neurovascular repair addresses interactions that are not captured by endothelial or neural endpoints alone. In streptozotocin-induced diabetic rat wounds, a sprayable hydrogel containing VEGF- and BDNF-mimetic peptides increased vascular and neural regeneration and produced a closer spatial association between CD31^+^ vessels and PGP9.5^+^ nerve fibers than the single-peptide formulations (Figure 3G) (57). The hydrogel also modified inflammatory and matrix features, indicating that its benefit arose from coordinated tissue effects and cannot be attributed to neural regeneration alone. Human association and *in vitro* evidence provide a more specific rationale for eccrine-directed repair: sudomotor innervation was associated with sweat function, and neural support increased glandular functional markers (46, 48). Selective sweat-gland reinnervation therefore remains a locally directed strategy with no demonstrated capacity to reverse arterial insufficiency, diffuse microvascular dysfunction, endothelial stress, peri-adnexal basement-membrane remodeling, or deep stromal arrest. Its potential benefit is likely to depend on adequate perfusion, viable tissue, controlled inflammation and infection, a repair-permissive extracellular matrix, and relief from repetitive mechanical stress. Offloading remains necessary to reduce such stress at the ulcer site (58).

Future studies should compare neural-targeted, vascular-targeted, and combined interventions while assessing more than nerve-fiber density. Evidence of broader Axis III recovery should include concordant improvement in sweat output and barrier function, local perfusion and endothelial reactivity, matrix remodeling, and wound closure. Improvement confined to innervation or sweating would demonstrate local target engagement without meeting the criteria for broader tissue recovery. Concordant recovery across these compartments would provide stronger evidence of broader tissue-level recovery. Even coordinated neurovascular recovery may not produce durable repair when keratinocyte, stromal, or endothelial populations remain proliferation-limited or senescent, which is the focus of Axis IV.

### 2.4 Axis IV—senescence-driven limits on repair

In DFUs, persistent arrest-associated states, sustained senescence-associated secretory activity, and reduced regenerative reserve may converge to constrain repair plasticity. In human DFU datasets, outcome-associated differences are most evident in basal keratinocyte cycling and differentiation-associated programs (59). Acute mouse-wound studies identified p21-high stromal and endothelial populations, whereas diabetic-mouse and human *ex vivo* experiments showed macrophage-to-fibroblast propagation of arrest-associated features (60, 61). p21/CDKN1A accompanies several forms of cell-cycle arrest, and its association with pathological senescence depends on the persistence and reversibility of arrest, accompanying SASP and damage features, and the lineage and temporal context (62). Within the chronic wound niche, sustained SASP and proteolytic outputs may reinforce Axis II inflammation and matrix injury. Hypoxic and neurovascular constraints described in Axis III, together with glycation-related and oxidative stress, may further restrict endothelial and regenerative-cell responsiveness. Diabetes also compromises adipose-derived stem cell (ADSC) viability, stress tolerance, and paracrine competence, narrowing the reserve available for cell-based support (63, 64). Together, these processes may limit Axis I re-epithelialization and Axis III vascular remodeling. Therapeutic strategies should therefore distinguish persistent maladaptive senescence-associated states from reversible arrest and transient repair-supportive senescence, selectively reduce pathological cells or outputs, and preserve repair-competent populations while restoring regenerative support where reserve is already compromised (4).

#### 2.4.1 Proliferative arrest across compartments

Single-cell and integrative transcriptomic analyses identify outcome-associated differences in proliferative competence across DFU cell populations, with the clearest separation occurring in basal keratinocytes. These differences do not by themselves identify pathological senescence. CDKN1A/p21 participates in protection from genotoxic stress as well as in cellular senescence (65). Quiescent cells can resume proliferation after appropriate stimulation, whereas senescent cells generally undergo prolonged arrest accompanied by secretory, damage-associated, and metabolic changes. Terminal differentiation and stress-associated arrest can also produce low-proliferation profiles without constituting senescence (62). A p21-high or Ki67-low snapshot therefore contributes to state characterization only when combined with evidence of persistence, functional irreversibility, accompanying SASP or damage features, and lineage and spatial context.

**Figure 4:**
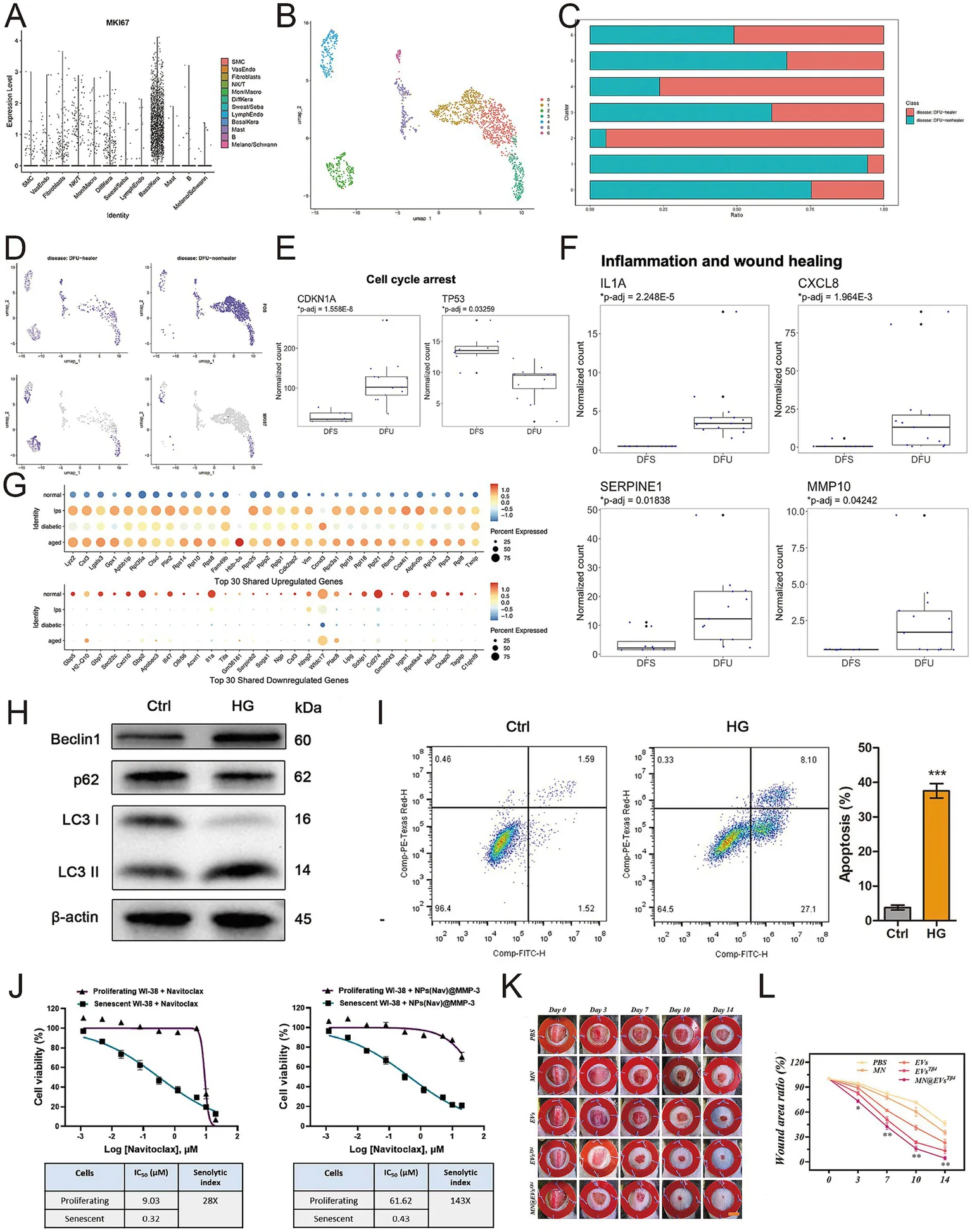
Proliferative arrest, senescence-associated programs, and experimental targeting in diabetic wound healing. **(A)** Distribution of MKI67 expression across major cell populations in a computational re-analysis of human foot-skin single-cell RNA-sequencing data from healthy individuals (*n* = 11), individuals with diabetes without foot ulcers (*n* = 8), patients with healing diabetic foot ulcers (*n* = 9), and patients with non-healing diabetic foot ulcers (*n* = 5) (59). **(B)** Uniform Manifold Approximation and Projection of seven re-clustered basal keratinocyte states, numbered 0–6, from human healing and non-healing diabetic foot ulcers (59). **(C)** Relative contributions of cells from healing and non-healing diabetic foot ulcers to basal keratinocyte clusters 0–6. Cluster numbers correspond directly to those in panel B, and each bar shows the relative group composition within a cluster rather than the absolute cell count (59). **(D)** Feature plots showing FOS and MKI67 expression in basal keratinocytes from human healing and non-healing diabetic foot ulcers (59). **(E)** Normalized bulk RNA sequencing counts of CDKN1A and TP53 in human diabetic foot ulcer-edge biopsies (*n* = 13 patients) and uninvolved diabetic foot skin (*n* = 8 patients) (65). **(F)** Normalized bulk RNA sequencing counts of IL1A, CXCL8, SERPINE1, and MMP10 in the same human tissue groups (65). **(G)** Top shared upregulated and downregulated genes in wound-fluid macrophages from aged, leptin receptor-deficient diabetic, lipopolysaccharide-treated delayed-healing, and normal-healing mouse wounds (*n* = 2 selected samples per group). Dot color represents average expression, ranging from low expression in blue to high expression in orange, and dot size represents the percentage of cells expressing each gene (66). **(H)** Western blot analysis of Beclin 1, p62, and microtubule-associated protein 1 light chain 3-I/II in cultured human adipose-derived stem cells under control and high-glucose conditions. Beta-actin was used as the loading control. (*n* = 3) (69). **(I)** Flow-cytometric analysis and quantification of apoptosis in cultured human adipose-derived stem cells under control and high-glucose conditions. (*n* = 3) (69). **(J)** Dose–response curves for proliferating and ionizing-radiation-induced senescent WI-38 human fibroblasts treated with free navitoclax or matrix metalloproteinase-3-responsive navitoclax-loaded nanoparticles. The accompanying tables show half-maximal inhibitory concentrations and the senolytic index, calculated as the half-maximal inhibitory concentration in proliferating cells divided by that in senescent cells. The senolytic index was 28 for free navitoclax and 143 for the nanoparticle formulation (72). **(K)** Representative wounds in streptozotocin-induced diabetic mice treated with phosphate-buffered saline, blank separable microneedle patches, unmodified adipose-derived stem cell extracellular vesicles, thymosin β4-engineered extracellular vesicles, or thymosin β4-engineered extracellular-vesicle-loaded separable microneedle patches (*n* = 5 mice per group). Images were obtained on days 0, 3, 7, 10, and 14. Scale bar, 5 mm (73). **(L)** Residual wound area over time in the five corresponding treatment groups in streptozotocin-induced diabetic mice (*n* = 5 mice per group) (73). *, **, and *** indicate *p* < 0.05, *p* < 0.01, and *p* < 0.001, respectively, as reported in the original source studies.

MKI67 expression is concentrated in basal keratinocytes (Figure 4A). Re-clustering resolved seven BasalKera states, numbered 0–6 in Figure 4B. The stacked bars in Figure 4C map directly to these numbered clusters and show the relative contribution of cells from healing and non-healing DFUs: clusters 0, 1, 3, and 5 were enriched for cells from non-healing DFUs, clusters 2 and 4 for cells from healing DFUs, and cluster 6 contained similar proportions. Consistent with this distribution, MKI67 expression was highest in clusters 2 and 4 and greater in healing DFUs, whereas FOS was highest in clusters 0 and 1 and greater in non-healing DFUs (Figure 4D). CDKN1A also varies along the inferred keratinocyte differentiation trajectory. FOS and JUNB were inferred to exert greater regulatory influence in non-healing samples, while external Cistrome data identified activator protein 1 peak signals at the CDKN1A locus. Mendelian randomization and summary-data-based Mendelian randomization analyses suggested an indirect metabolic connection involving the *α*-ketobutyrate/pyruvate ratio, although the direct CDKN1A–DFU relationship was not significant (*p* > 0.05). CDKN1A consequently marks an outcome-associated proliferative and differentiation program whose causal contribution to non-healing remains unresolved (59).

Heterogeneity in p21-associated states extends beyond the epidermis. Spatially resolved analysis of acute mouse wounds distinguished p21-high from p16-high populations. The p21-high population included fibroblasts, endothelial cells, M1 macrophages, and keratinocytes and carried pro-inflammatory and tissue-remodeling transcriptional programs that were not similarly enriched in p16-high cells. Most proliferating wound-edge keratinocytes lacked the markers used to identify the p21-high population, and genetic clearance of p21-high cells accelerated closure in female mice without producing a comparable benefit in males (60). In diabetic-mouse macrophage-conditioned medium and human-fibroblast experiments, macrophage-derived CXCR2 ligands induced nuclear p21 and other senescence-associated features in dermal fibroblasts, linking Axis II inflammatory signaling to stromal arrest-associated programs (61). Repair-limiting programs may therefore spread across lineages. Their relevance to senescence-directed therapy depends on lineage, spatial location, persistence, and accompanying functional features.

#### 2.4.2 Senescence-associated inflammation and matrix remodeling

Persistent cell-cycle arrest becomes functionally consequential when it is coupled to secretory and matrix-remodeling outputs. Bulk RNA sequencing of human DFU wound-edge biopsies relative to uninvolved diabetic foot skin showed increased CDKN1A and reduced TP53 expression (Figure 4E), together with coordinated induction of IL1A, CXCL8, IGFBP2, MMP10, SERPINE1, and TGFA (Figure 4F) (65). At the tissue level, these changes reveal a coordinated CDKN1A/SASP network without resolving its cellular sources or determining which cells have entered an irreversible senescent state.

Mechanistic and single-cell studies connect this network to immune-stromal signaling. Diabetic macrophage-derived CXCR2 ligands induce nuclear p21 and other senescence-associated features in dermal fibroblasts, and local CXCR2 antagonism improves repair in diabetic mouse and human *ex vivo* wound models (61). Single-cell analysis of wound-fluid macrophages identified a delayed-healing program shared across aged, diabetic, and lipopolysaccharide-exposed murine wounds, including induction of ribosomal-stress Rpl/Rps transcripts and Ccl2 together with reduced Tnfaip3, Junb, and Il1r2 expression (Figure 4G) (66). Computational integration further associated non-healing fibroblast states with senescence-related, complement, and dysregulated ECM-interaction programs (67). SASP-related cytokines and proteases may consequently sustain leukocyte recruitment, matrix degradation, keratinocyte stress, and endothelial instability. Through these effects, Axis IV may amplify Axis II inflammation while further constraining the reparative programs of Axes I and III.

Senescence-associated states can also support repair in a time-dependent manner. In normal acute mouse wounds, transient senescent fibroblasts and endothelial cells accumulated during repair, secreted PDGF-AA, supported granulation tissue formation and wound closure, and subsequently declined. Genetic or pharmacological depletion of these cells delayed healing, and PDGF-AA partially rescued the repair defect (68). In a separate acute-wound study, p21-high and p16-high populations were distinct; genetic clearance of p21-high cells attenuated NF-κB-associated signaling and accelerated closure in female mice without producing a comparable benefit in males (60). Marker positivity and cell depletion therefore carry different biological consequences across wound phases, cell populations, and experimental contexts.

The contrast among these studies distinguishes transient, repair-supportive senescence-associated programs from persistent states that may become maladaptive under impaired clearance and continuing chronic-wound stress. Reasonable candidates for senescence-directed therapy would remain stably proliferation-arrested within chronic DFU niches and simultaneously exhibit SASP or proteolytic output, macromolecular or cellular damage, altered metabolism, or apoptosis resistance, together with lineage- and spatially resolved evidence of continued contribution to non-healing (62). Isolated p21 positivity, low Ki67 expression, or another single senescence-associated marker does not meet this threshold. The therapeutic aim is to reduce persistent pathological senescence-associated burden while preserving transient repair-supportive cells and lineage-competent progenitors.

#### 2.4.3 Short-lived regenerative support

ADSCs provide an experimentally tractable source of regenerative support whose therapeutic activity depends on viability, stress tolerance, and paracrine competence. ADSCs isolated from patients with type 2 diabetes already exhibit reduced proliferation and viability, impaired antioxidative protection, mitochondrial dysfunction, and altered secretory activity, narrowing their capacity to support cell-based repair (63). ADSCs consequently provide a model of therapeutic regenerative reserve, while endogenous epidermal, stromal, and vascular progenitor populations require separate lineage-specific assessment.

Advanced glycation end products can rapidly reduce ADSC viability through receptor for advanced glycation end products–p38-associated apoptotic signaling. AGE-HSA increased apoptosis and caspase-3 activity in human ADSCs within 24 h, demonstrating rapid apoptotic injury under experimental exposure without establishing a senescent state (64). In cultured human ADSCs exposed to high glucose, single-cell multi-omics and experimental validation identified a c-Myb–AURKA-associated program involving autophagy, metabolic adaptation, and cell survival. High-glucose exposure increased Beclin1 expression and LC3-I-to-LC3-II conversion (Figure 4H) while also increasing apoptosis (Figure 4I) (69). Early autophagic and metabolic responses and subsequent loss of viability are related components of the stress response, but none alone establishes irreversible senescence. ADSCs derived from patients with type 2 diabetes also show oxidative stress, mitochondrial fragmentation, impaired antioxidative protection, reduced secretion of pro-repair factors including VEGF and CXCL12, and altered expression of selected extracellular-vesicle miRNAs (63).

Within the DFU niche, excessive MMP activity can degrade ECM components and growth-factor signals required for effective paracrine repair (70). Hypoxic constraints described in Axis III may further reduce cell survival and responsiveness after delivery. The diabetic environment consequently limits regenerative support at both the cell-source and recipient-niche levels, making regenerative-cell optimization and correction of local proteolytic and hypoxic constraints complementary requirements. This reduced reserve also makes residual repair competence an essential safety and efficacy endpoint for senotherapy. Decreases in p21, p16, senescence-associated β-galactosidase, or SASP signals would not establish therapeutic success unless basal/progenitor, fibroblast, endothelial, and delivered-cell functions are maintained or restored.

#### 2.4.4 Reducing senescence and restoring regenerative signals

Therapeutic decisions in Axis IV require translation of the pathological-state criteria described earlier into lineage-resolved, *in situ* target qualification. p21 expression alone cannot distinguish transient arrest, repair-supportive senescence-associated activity, terminal differentiation, or a persistent pathological state. A candidate population for senolysis would therefore require convergent evidence of chronic cell-cycle arrest, SASP or proteolytic activity, macromolecular damage, metabolic remodeling, and apoptosis resistance within a defined lineage and spatial niche. Depending on specimen handling, an in situ panel could combine SA-beta-gal activity or lipofuscin accumulation with p16/p21, proliferation markers, loss of LMNB1, SASP proteins, and lineage markers (62). At the wound edge, such qualification must explicitly distinguish the intended pathological population from basal and progenitor cells that retain the capacity for cell-cycle re-entry, migration, and differentiation.

Even after a pathological population has been identified, its suitability for senolysis depends on the phase of wound repair. In acute mouse wounds, transient senescent fibroblasts and endothelial cells contributed to repair through PDGF-AA secretion, and their genetic or pharmacological depletion delayed closure (68). In a db/db-high-fat diet mouse model, systemic ABT263/navitoclax administration was initiated before wound creation and subsequently improved closure (71). Genetic clearance of p21-high cells in acute wounds produced context- and sex-dependent effects (60). These models establish treatment timing as a determinant of biological effect, but they do not define when a persistent state in an established human DFU becomes both dispensable and safely removable. Senolysis should therefore be considered after chronic persistence and pathological output have been demonstrated, with particular caution during early inflammatory and proliferative phases when transient arrest-associated programs may still contribute to repair.

Once the target state and treatment window have been specified, delivery design determines whether exposure can be sufficiently restricted to reduce systemic and off-target toxicity. Local, short-course, or microenvironment-gated administration may reduce circulating exposure and limit toxicity to non-target cells. In irradiation-induced senescent WI-38 fibroblasts, an MMP-3-responsive navitoclax nanodevice preserved senolytic activity while reducing toxicity to proliferating cells. The senolytic index, calculated as the half-maximal inhibitory concentration in proliferating cells divided by the half-maximal inhibitory concentration in senescent cells, increased from 28 for free navitoclax (9.03/0.32 μM) to 143 for NPs(Nav)@MMP-3 (61.62/0.43 μM), corresponding to an approximately 5.1-fold increase (Figure 4J) (72). This result demonstrates improved exposure selectivity in a defined *in vitro* system. Biological selectivity in DFU would additionally require MMP-3 or another gating signal to be enriched in the intended pathological population, absent or sufficiently low in repair-competent cells, and linked to preserved basal/progenitor function after treatment. Current evidence has not established such selective elimination in human DFU, making lineage-resolved safety testing a prerequisite for translation.

Exposure control alone cannot determine whether the targeted cells should be eliminated or functionally rescued; that choice depends on the persistence, reversibility, and lineage identity of the pathological state. Senolysis is most defensible when a lineage-resolved population is chronically arrested, SASP-active, damage-bearing, and apoptosis-resistant. Genetic or pharmacological removal of p21-high cells belongs to this cell-depletion or senolytic-like category and is distinct from senomorphic intervention (60). When arrest reversibility or cellular identity remains uncertain, reducing pathological secretory output or restoring function offers a more conservative route. Local CXCR2 antagonism attenuated macrophage-to-fibroblast senescence-associated signaling and improved repair in diabetic mouse and human *ex vivo* wound models (61). In db/db mouse wounds treated with Tβ4-engineered ADSC extracellular-vesicle microneedle patches, the intervention reduced senescence-associated β-galactosidase- and p16/p21/p53-associated signals, improved fibroblast and endothelial function, and accelerated diabetic wound repair through PTEN/PI3K/AKT-linked functional restoration (Figures 4K,L) (73). Neither intervention has yet established reversal of irreversible senescence or preservation of endogenous progenitors, and the physiological roles of CXCR2 in leukocyte recruitment, migration, and angiogenesis make dose, timing, and lineage-specific safety central to further evaluation.

Removing or suppressing a pathological state may be insufficient when the regenerative reserve is already depleted. Diabetic ADSCs exhibit c-Myb-AURKA-associated autophagic and metabolic adaptation, providing a mechanistic basis for preconditioning or engineering before delivery (69). In FGF21-engineered ADSCs and diabetic mouse wounds, the intervention increased resistance to ferroptosis and oxidative stress through SIRT1/NRF2/GPX4-associated signaling and improved repair (74). Among cell-free approaches, a single-center randomized trial reported shorter time to DFU closure following treatment with Wharton’s jelly mesenchymal stem cell-derived exosomes (75). ADSC-derived exosomes also enhanced epidermal autophagy and re-epithelialization in diabetic mice and implicated NAMPT-related autophagy-NAD signaling (76). Regenerative support may therefore complement state-directed treatment, although the preliminary clinical signal requires independent confirmation, and extracellular vesicle translation will depend on standardized product identity, functional potency assays, and reproducible activity within the proteolytic, hypoxic, and mechanically stressed DFU niche.

Therapeutic success has two inseparable endpoints: reduction of the persistent pathological state and preservation or restoration of repair capacity. Studies should quantify lineage-resolved basal/progenitor abundance, cell-cycle re-entry, clonogenic or organoid-forming competence, re-epithelialization and durable closure, fibroblast and endothelial reparative function, and platelet or other hematologic toxicity. These endpoints should be compared across local and systemic exposure and across early and chronic treatment windows. Axis II inflammatory, CXCR2, and proteolytic signals and Axis III hypoxia, glycation, oxidative stress, and neurovascular dysfunction may sustain arrest-associated states (61, 64, 70). In turn, persistent SASP and proteolytic activity can reinforce Axis II inflammation and constrain Axis I epithelial repair and Axis III vascular remodeling. A clinically credible strategy must therefore qualify the target state, match treatment to wound stage, control exposure, reserve cell depletion for well-resolved pathological populations, restore regenerative capacity when needed, and monitor both sides of this therapeutic balance. Longitudinal human studies remain necessary to determine whether this sequence can interrupt the complete cross-axis maintenance loop.

## 3 Axis-guided translation to precision DFU therapy

### 3.1 Stratification and prognostic scores

Multi-parameter axis scores may be more informative than single biomarkers in heterogeneous DFUs, particularly because individual molecular markers can show limited generalizability across cohorts. For example, a multicenter consortium study found that tissue c-Myc and phosphorylated glucocorticoid receptor did not predict DFU healing, highlighting the difficulty of relying on isolated biomarkers for outcome stratification (77). By integrating cell-state composition, signaling activity, and cross-compartment coupling, axis scores may provide a more biologically integrated composite metric once validated.

Clinical deployment requires staged empirical validation before routine use. A binary “high/low” schema across the four axes could serve as an interpretable first approximation, with thresholds derived from outcome-annotated cohorts. For example, an Axis I-high/Axis II-high profile may indicate impaired barrier recovery coupled with inflammatory-proteolytic feedback, whereas an Axis I-high/Axis III-high profile may reflect epidermal failure under ischemic constraints. Axis II-high/Axis IV-high and Axis III-high/Axis IV-high profiles may indicate senescence-amplified inflammation or neurovascular failure with limited regenerative reserve, respectively. These profiles can generate testable treatment hypotheses and should be integrated with established clinical staging systems, such as wound, ischemia, and foot infection, through retrospective and prospective validation.

The feasibility of this strategy is supported by recent high-dimensional studies in other diseases. In oncology, BayesPrism used patient-derived single-cell RNA-seq as prior information to infer cell-type composition and cell-type-specific gene expression from large bulk RNA-seq cohorts, enabling associations between inferred cellular states and clinical outcomes across multiple tumor types (78). In rheumatoid arthritis, single-cell-defined cell-type abundance phenotypes were mapped to lower-resolution clinical cohort data, including bulk RNA-seq, and were associated with treatment response, supporting the translational value of cell-state-based tissue stratification (79). These studies provide methodological precedents for linking single-cell-derived tissue states with outcome-annotated clinical cohorts, while DFU-specific predictive utility remains to be tested.

A practical retrospective validation strategy would project single-cell-defined DFU axis signatures onto publicly available DFU bulk RNA-seq or microarray cohorts with annotated healing outcomes. Cell-state markers derived from DFU single-cell atlases could be condensed into four axis-level signatures and quantified in bulk datasets using cell-state deconvolution methods, such as CIBERSORTx, MuSiC, BisqueRNA, or BayesPrism, or single-sample signature scoring approaches, such as gene set variation analysis or singscore. The resulting axis activities could be tested for prediction of clinically meaningful outcomes, including 12-week healing, 4-week percent area reduction, recurrence risk, or amputation-free wound closure, using logistic regression, Cox models, regularized regression, or random-forest classifiers. Model performance should be evaluated by discrimination, calibration, internal cross-validation, external dataset testing where available, and decision-curve analysis, with incremental value assessed against established clinical predictors such as Wound, Ischemia, and Foot Infection stage and early percent area reduction. This validation framework would clarify whether the proposed four-axis model captures reproducible prognostic information beyond conventional single biomarkers.

### 3.2 Combination therapy by axis coupling

Therapeutic combinations in DFU should follow axis coupling principles to address high-impact, cross-compartment dependencies. Single-cell atlases show that non-healing wounds sustain a self-perpetuating inflammatory state, where parallel feedback pathways compensate for single-pathway inhibition. Mechanistically, Axis II exacerbates Axis I through protease-driven matrix degradation, Axis III amplifies Axis II via hypoxia-inflammation loops, and Axis IV decreases global tissue plasticity. In outcome-stratified human DFU profiling, non-healing ulcers showed concurrent enrichment of inflammatory myeloid–fibroblast programs, endothelial dysfunction, and reduced keratinocyte differentiation trajectories (4).

Clinical adjuncts often increase closure probability but do not move the wound out of this stable, non-healing state if upstream drivers persist. In the randomized multicenter Graftskin trial involving patients with noninfected neuropathic DFUs, living skin equivalents increased 12-week closure compared with standard care, although a substantial proportion of treated ulcers remained open (80). In a separate randomized trial involving patients with DFUs, negative-pressure wound therapy increased complete closure compared with advanced moist wound therapy, but most wounds in both groups remained unhealed (81). These clinical results demonstrate incomplete efficacy of individual modalities; they do not identify persistent hypoxia or inflammation as the proven cause of treatment non-response.

These dependencies support a staged therapeutic strategy. Stage 1 should prioritize alleviating upstream inhibitory constraints by jointly addressing Axis II and Axis III. Coupling between Axis II and Axis I explains why growth factors underperform in protease-rich environments: the immune-stromal lock-in maintains cytokines that degrade the provisional matrix required for keratinocyte migration. Evidence supports targeting this link; in splinted db/db mouse wounds, matrix-binding IL-1 receptor antagonism accelerated re-epithelialization and closure (23). Clinically, sucrose octasulfate dressings, which are designed to sequester excess proteases, significantly increase closure rates in neuroischemic ulcers by protecting endogenous growth factors (43). Simultaneously, Axis III and Axis II coupling requires resolving hypoxic constraints. Adjunctive hyperbaric and topical oxygen therapies have shown efficacy in randomized trials, validating oxygenation as a prerequisite for breaking the hypoxia-inflammation cycle (52, 53).

Stage 2 focuses on restoring cellular responsiveness once the microenvironment permits. Reducing protease burden and hypoxic stress enables pro-repair signaling to result in measurable re-epithelialization. In high-glucose human keratinocytes and diabetic mouse wounds, epigenetic inhibition reduced MMP9-associated migratory impairment and improved re-epithelialization (14). This principle of “state correction” also applies to the vasculature; in diabetic endothelial-cell experiments and ischemic mouse wounds, PLCγ2-targeted epigenetic editing restored VEGF responsiveness and perfusion (55). Therefore, epidermal and vascular interventions should be sequenced either after or concurrently with the alleviation of inhibitory environmental constraints.

Stage 3 addresses the regenerative exhaustion of Axis IV. When proliferative arrest persists, therapeutic sequencing becomes critical. Senescence-associated programs have phase-dependent effects: evidence from acute mouse wounds indicates that transient senescence supports repair via PDGF-AA (68), whereas persistent accumulation drives chronic inflammation (60). A rational strategy combines senotherapy with regenerative replenishment. Clearing senescent cells reduces the inflammatory load, creating a window for cell- or extracellular vesicle-based therapies. One single-center randomized clinical study in patients with DFUs reported shorter time to closure after treatment with Wharton’s jelly mesenchymal stem cell-derived exosomes (75). Translational feasibility is further enhanced by microenvironment-responsive delivery systems that limit off-target toxicity (72).

In summary, this staged framework prioritizes a durable transition to healing. Combination therapies can systematically dismantle the coupled mechanisms sustaining the non-healing state by first attenuating the inflammatory-hypoxic constraints of Axis II and III, then restoring the cellular responsiveness of Axis I, and finally replenishing the regenerative reserve of Axis IV.

### 3.3 Trials: longitudinal single-cell/spatial endpoints

Integrating longitudinal single-cell and spatial profiling into DFU trials shifts evaluation from simple closure to mechanism-informative studies, enabling direct quantification of axis engagement and tissue-state transitions. Relying on closure alone cannot distinguish between inadequate target engagement and the targeting of non-rate-limiting pathways. To capture these molecular dynamics, we need a practical framework that anchors profiling to standard-of-care debridement using a three-point sampling strategy: a post-debridement baseline, an early pharmacodynamic readout (e.g., days 7–14), and an outcome-linked specimen at the trial endpoint. Feasibility is supported by protocols that recover high-quality single-cell data from debridement-derived tissue, allowing for serial monitoring with minimal incremental burden (6). Critically, the sampled compartment must match the mechanistic target, using wound-edge epidermis for keratinocyte differentiation, the wound bed for immune-stromal interactions, and spatially preserved biopsies for neurovascular restoration.

Building on this sampling strategy, endpoints must be prespecified as quantitative tissue-state transitions. Key spatial metrics include the transition from disorganized inflammatory aggregates to structured granulation tissue, the restoration of neurovascular recoupling, and the re-establishment of epithelial barrier integrity, using outcome-stratified molecular atlases as the empirical framework for clinical grading (4). Furthermore, spatiotemporal designs are critical; averaging “edge” and “bed” molecular programs obscures spatially restricted mechanistic failures unique to the non-healing niche (82). In addition to spatial data, single-cell endpoints focus on directionally constrained readouts mapped to the four axes, specifically compositional shifts and axis activity scores. Evaluating these readouts within an early on-treatment window enables the identification of responders before macroscopic changes occur, a predictive capacity shown in inflammatory skin disease trials (83).

Ultimately, integrating these readouts facilitates a structured failure analysis that distinguishes regimen failure from unaddressed clinical constraints like offloading or infection. Successful deployment across centers requires rigorous standardization, with core gene sets and spatial marker panels specified *a priori*, and integration pipelines using reproducible computational frameworks to control for batch effects and cross-site variability.

## 4 Discussion

Single-cell and spatial profiling support a view of DFU non-healing as a dynamic, spatially heterogeneous condition sustained by four interacting mechanistic axes. This framework moves beyond cell-type enumeration by identifying recurrent state alterations and transition failures that may be therapeutically tractable. Immune–stromal feedback (Axis II) and neurovascular dysfunction (Axis III) may reinforce epidermal arrest (Axis I), limiting progression from inflammatory activation to barrier reconstitution. Concurrent proliferative arrest and persistent senescence-associated programs across multiple lineages (Axis IV) further constrain regenerative plasticity. The coexistence of these processes may limit responses to interventions directed at a single pathway.

Translating this multi-axis framework requires an operational triad—axis-based stratification, rational combination therapy, and longitudinal mechanistic readouts. Axis-specific constraints define a coupled state space in which therapeutic success depends on alleviating upstream inhibitory loops either before or concurrently with inducing regeneration. For example, reducing inflammatory-proteolytic burdens while resolving hypoxia creates a permissive microenvironment that allows epidermal and vascular repair programs to proceed. Therefore, clinical trials must shift from simple closure endpoints to designs that quantify early pharmacodynamic changes in axis scores and spatial architecture, allowing investigators to distinguish between effective target engagement and mechanistic non-response.

Although longitudinal molecular readouts can distinguish early target engagement from mechanistic non-response, they do not by themselves establish causal direction. Most current human DFU atlases remain cross-sectional and correlational, while snapshot profiling and computational inference cannot determine the temporal order of cross-compartment changes. Serial, spatially resolved sampling at baseline, early after a prespecified intervention, and at outcome could establish whether shifts in inflammatory myeloid states precede recovery of fibroblast repair programs or vice versa, but temporal precedence alone would not prove causality. Directionality requires compartment-specific perturbation and rescue. Recovery of fibroblast remodeling after selective inhibition of inflammatory myeloid recruitment or activation, without direct fibroblast manipulation, would support a myeloid-to-stromal contribution. Conversely, reduced subsequent immune recruitment or retention after selective normalization of fibroblast state or chemokine output, without direct myeloid intervention, would support a stromal-to-myeloid contribution. Reversing the cross-compartment effect by reinstating the perturbed signal would strengthen causal attribution. The timing, magnitude, and durability of cross-compartment rescue would help distinguish the initiating event from the dominant maintenance node. If either single-compartment intervention produces only partial or transient recovery, whereas dual or sequential intervention yields durable resolution and matrix remodeling, this would support a self-maintaining immune-stromal loop. Such studies require cautious model-to-human validation because chronic human ulcers differ from experimental wounds in immune composition, ischemic burden, microbial exposure, and tissue architecture.

In addition to cross-cohort variability and unresolved causality, several technology-inherent limitations should be considered when interpreting the single-cell and spatial evidence summarized here. Preparation of viable single-cell suspensions can selectively deplete fragile or dying cells, alter the apparent abundance of keratinocyte, endothelial, and immune subsets, and introduce *ex vivo* stress-response signatures that may resemble inflammatory activation. Enzymatic digestion time, temperature, and mechanical dissociation can further affect cell recovery across epidermal, vascular, stromal, and immune compartments. Dead or dying cells that remain in the suspension may contribute ambient RNA or stress-associated transcripts, whereas stringent viability filtering may remove biologically relevant damaged-cell states. Sequencing depth and cell capture rate also influence the detection of rare transitional states. Nuclei-based approaches may reduce some dissociation-related losses but provide a different molecular readout from whole-cell RNA sequencing. Spatial transcriptomic assays retain tissue architecture, yet their resolution can be insufficient to assign signals to individual cells in dense granulation tissue, where immune cells, fibroblasts, endothelial structures, and migrating keratinocytes may occupy the same capture area. In addition, ligand-receptor inference and trajectory reconstruction remain computational predictions rather than direct evidence of signaling or causality. Future studies should combine standardized sampling, viability-aware processing, higher-resolution spatial platforms, *in situ* validation, protein-level confirmation, and perturbation experiments to distinguish robust disease programs from technical artifacts (11–13).

Ultimately, the path to clinical implementation involves distilling complex atlases into standardized, assayable panels compatible with routine debridement specimens. These tools should yield interpretable axis scores to guide initial modality selection and adaptive treatment sequencing. By harmonizing prospective cohorts with causal validation, this four-axis framework provides a foundation for transforming DFU care from empirical trial-and-error to mechanism-guided precision intervention.
